# Community‐based health programme for nurses and midwives: A mixed methods evaluation

**DOI:** 10.1111/jan.16336

**Published:** 2024-07-30

**Authors:** Rebecca Jarden, Aaron Jarden, Helena Bujalka, Tracey Weiland, Naomi Brockenshire, Glenn Taylor, Marie Gerdtz

**Affiliations:** ^1^ Department of Nursing, Melbourne School of Health Sciences, Faculty of Medicine, Dentistry and Health Sciences The University of Melbourne Carlton Victoria Australia; ^2^ Austin Health Heidelberg, Melbourne Victoria Australia; ^3^ Centre for Wellbeing Science, Faculty of Education The University of Melbourne Parkville, Melbourne Victoria Australia; ^4^ Melbourne School of Population and Global Health The University of Melbourne Carlton, Melbourne Victoria Australia; ^5^ Nursing and Midwifery Health Program – Victoria Cremorne Victoria Australia

**Keywords:** case management, health, health service, midwife health, model of care, nurse health, programme, service evaluation, well‐being

## Abstract

**Aim:**

To evaluate a community‐based psychological health and well‐being programme for nurses and midwives.

**Design:**

Mixed methods programme evaluation.

**Methods:**

Four studies were included: observational descriptive study (cross‐sectional survey) of the health, well‐being and experiences of previous programme participants (Study 1); observational exploratory prospective cohort study (longitudinal survey) of health, well‐being and experiences of participants who engaged in the programme from 2020 to 2023 (Study 2); qualitative descriptive study (interviews) of experiences and perceptions of nurses and midwives who have engaged with the programme as participants or clinicians (Study 3); observational descriptive study (cross‐sectional survey) of experiences and perceptions of programme stakeholders (Study 4). Surveys included validated measures. Data were collected online. Descriptive, repeated measures and thematic analyses were conducted.

**Results:**

One‐hundred and fifteen participants completed Study 1: 20% (*n* = 23) reported stress in the severe‐to‐extremely severe category; 22% (*n* = 25) reported psychological distress in the moderate‐to‐severe category. Thirty‐one programme participants were followed in Study 2: the effect of the programme on participant well‐being over time was not significant. Sixteen programme participants and eight programme clinicians were interviewed (Study 3). Experiences of nurses and midwives engaging with the programme were highly positive and strong attributes of the programme included (1) shared professional experience of clinicians and participants which supported a common language and facilitated understanding, and (2) effective programme leadership, and autonomy and flexibility in the clinicians' role which enabled and supported a positive working experience. Thirty‐nine broader stakeholders participated in a cross‐sectional survey (Study 4). All stakeholders reported high satisfaction with the programme. Participants considered the programme being ‘by nurses and midwives, for nurses and midwives’ critical to the programme's success and value.

**Conclusions:**

The community‐based psychological health and well‐being programme developed, led and delivered by nurses and midwives, for nurses and midwives, was a highly valued resource.

**Impact:**

- Levels of stress and burnout in the health workforce are high.
- A community‐based psychological health and well‐being programme for nurses and midwives was found to be an important and highly valued resource for nurses and midwives.
- A programme delivered by nurses and midwives, for nurses and midwives, was considered critical to programme success.
- Programme leadership, and autonomy and flexibility in the programme clinicians' roles, facilitated and supported a positive working experience for programme clinicians.

**Implications for the Profession and Patient Care:**

Quality and safety in patient care is directly impacted by the well‐being of nurse and midwives. A community‐based psychological health and well‐being programme for nurses and midwives was found to be an important and highly valued resource for nurses and midwives.

**Reporting Method:**

Survey findings were reported according to STROBE (von Elm et al. in *Lancet*, 370:1453–1457, 2007) and qualitative findings according to COREQ (Tong et al. in *International Journal for Quality in Health Care*, 19(6):349–357, 2007).

**Patient or Public Contribution:**

No patient or public contribution.

**What does this paper contribute to the wider global clinical community?**

People's lives depend on access to a strong and capable health workforce. Yet levels of stress and burnout in the health workforce have never been so evident, and nurses and midwives are leaving the profession early. A community‐based psychological health and wellbeing programme delivered by nurses and midwives, for nurses and midwives, was highly valued by stakeholders.

## 1 INTRODUCTION

Occupational stress and burnout are longstanding and complex global problems in the nursing and midwifery professions, predicted by high workload, low staffing levels, long shifts and low autonomy. Retaining nurses and midwives and students, entering these professions is crucial amid attrition in entry‐to‐practice education programmes and early career health professionals, and increasing rates of retirement. A range of interventions are proposed to address occupational stress and burnout. Community‐based psychological health and well‐being programmes are one such intervention to support and consequently retain nurses and midwives.

## 2 BACKGROUND

Maintaining a health workforce to meet increased health care demand is a widespread challenge (Burns et al., 2020; World Health Organization, 2020b). For individuals, work contributes to mental health, recognized in the World Health Organization's definition of mental health (World Health Organization, 2013, p. 6). For organizations, the well‐being of health workers influences performance in relation to care, conduct and leave (Brunetto et al., 2013; Ray‐Sannerud et al., 2015). Australian nurses and midwives reported higher levels of anxiety, depression and stress during the COVID‐19 pandemic than the general Australian adult norms (Holton et al., 2021), reflecting broader international findings (Mharapara et al., 2022) and the longstanding negative impacts of workplace adversity on well‐being (Moran et al., 2023). These negative impacts, reflected in ill‐health such as burnout, have been the focus of considerable research over the past decades, with demonstrable patterns of individual characteristics such as age, experience and living situation (Suleiman‐Martos et al., 2020), and adverse job characteristics such as high workload, low staffing levels, long shifts and low control (Dall'Ora et al., 2020; Suleiman‐Martos et al., 2020).

Investigating the impact of COVID‐19 on health workers is ongoing (Waters et al., 2021), and these studies have begun to explore stress, anxiety, distress and fear (Holton et al., 2021; Hu et al., 2020) with many recommendations (Maben & Bridges, 2020; World Health Organization, 2020a) and interventions to support mental health. Several reviews of interventions to support the psychological health and well‐being of health workers have been published in the past 5 years such as those for nurse leaders (Häggman‐Laitila & Romppanen, 2018), multi‐component interventions (Hendriks et al., 2020), physical health and well‐being interventions (Fadel et al., 2023; Shiri et al., 2023), psychological interventions (van Agteren et al., 2021), positive psychology interventions (Donaldson et al., 2019), positive psychological interventions for subjective and psychological well‐being (Koydemir et al., 2021), web‐based psychological interventions delivered in the workplace (Carolan et al., 2017) and interventions during pandemics (Pollock et al., 2020; Robins‐Browne et al., 2022). In a review of community‐based programmes for people experiencing mental illness in the Australian context, three categories were reported: therapeutic, case management or lifestyle. Therapeutic and case management categories were found most effective in reducing psychiatric symptoms (O'Donnell et al., 2020).

### 2.1 Context: A community health programme for nurses and midwives

The current evaluation is of a Health Program (hereafter, the Program) established in the early 2000s and based in the community in Victoria, Australia. The Program works within a recovery focused counselling framework and services are provided exclusively by experienced nurses and midwives. These services are designed for nurses and midwives, and nursing and midwifery students, experiencing issues impacting their health and well‐being. The Program aims to promote health, well‐being and resilience, and reduce the risks to those who use nursing and midwifery services. Nurses and midwives delivering the services operate within a health and well‐being framework incorporating prevention, intervention and restoration. A case management model of care is used, and nurses and midwives are either referred or self‐refer. The service is free, confidential and independent of health care organizations. Nurses, midwives and students commonly access the Program after experiencing sensitive health issues related to their mental health, substance use, family violence or any issue impacting their health and well‐being.

Following referral to the Program, potential Programme participants' first point of contact is a professional, trained in working with people presenting with psychological problems, such as distress and providing reassurance. Program participants are then assigned to a case manager who is a nurse or midwife, who assesses the participant, and provides an individual care plan and support. This care plan and support may include referral, treatment, liaison with employer and support to re‐enter the workplace as appropriate to the individual participant. Investigating psychological health and well‐being characteristics and experiences of people engaging with this programme is important in establishing a baseline understanding of how the Program is working, for whom and why. This understanding will provide novel insights in developing future programmes and a baseline for future interventions to strengthen community‐based health and well‐being programmes for health professionals.

## 3 THE STUDY

### 3.1 Objectives and research questions

The objectives of this evaluation were to determine the (1) psychological health and well‐being characteristics of nurses and midwives engaging in the Program, (2) effectiveness of the case management model on the well‐being of nurses and midwives, (3) experiences and perceptions of nurses and midwives engaging in the Program as participants or service providers and (4) experiences and perceptions of broader stakeholders of the Program. To this end, we describe previous participants', referrers' and referral services' perceptions and experiences of their engagement with the Program, and measure new participants' levels of health and well‐being on engagement with the Program, at exit from the Program and at 12 months following initial engagement.

The four research questions (RQs) were as follows:

RQ1: What are the psychological health and well‐being characteristics of nurses and midwives engaging in the Program?
RQ2: What is the effectiveness of the case management model on the well‐being of nurses and midwives?
RQ3: What are the experiences and perceptions of nurses and midwives engaging in the Program as participants and clinicians?
RQ4: What are the experiences and perceptions of broader Program stakeholders?

## 4 METHODOLOGY

This evaluation was underpinned by the philosophical paradigm of pragmatism to enable the focus to be on ‘what works’ and determine solutions that best meet the needs and purpose (Creswell, 2013), and was guided by the World Health Organization (2007) recommendations for evaluation of mental health policies and plans, Centres for Disease Control and Prevention (CDC; USA Department of Health and Human Services Centers for Disease Control and Prevention, 2011). Survey findings were reported according to STROBE (von Elm et al., 2007) and qualitative findings according to COREQ (Tong et al., 2007).

### 4.1 Design

To address the research questions, a concurrent triangulated mixed methods programme evaluation was conducted using both qualitative and quantitative research methods to draw on the strengths of both and to address the complexity of the questions (Creswell & Plano Clark, 2007). Study 1 was an observational descriptive study (cross‐sectional survey) of previous programme participants from 2006 until end of 2020. Study 2 was an observational exploratory prospective cohort study (longitudinal survey) of participants who engaged in the programme from 2020 to 2023. Study 3 was a qualitative descriptive study (interviews) of nurses and midwives who have engaged with the programme as participants or clinicians from 2006 until 2022. Study 4 was an observational descriptive study (cross‐sectional survey) of key programme stakeholders in 2022.

**FIGURE 1:**
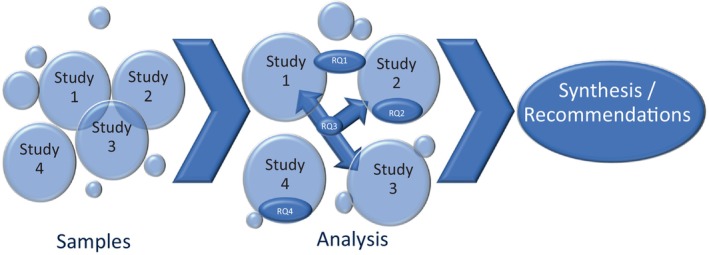
Concurrent triangulated mixed methods evaluation design.

Data analysis and study findings were either reported individually or synthesized to address the four RQs and meet overarching objectives. To address RQ1, items from Study 1 and Study 2 were analysed, synthesized and reported. To address RQ2, Study 2 findings were reported. To address RQ3, items from Study 1 and Study 2, and all Study 3 interview data were analysed and reported, then findings were synthesized. To address RQ4, Study 4 findings were analysed and reported. Triangulation of studies, including sampling and analysis, is illustrated in Figure 1.

### 4.2 Methods

#### 4.2.1 Data collection and instruments

The three studies involving online surveys (studies 1, 2 and 4) used REDCap (Research Electronic Data Capture) electronic data capture tools hosted at the University of Melbourne to both collect and manage data (Harris et al., 2019).

For studies one and two, the survey assessment battery comprised up to 180 items (approximately 20 min) which captured the variables of interest. The first question in the survey asked respondents to indicate whether they had most recently participated in the Program prior to 2020 or from 2020 onwards; those who indicated as the former participated in the cross‐sectional branch of the study, completing the survey at a single timepoint; those who indicated they had participated from 2020 onwards participated in the longitudinal branch of the study, which involved completion of the survey at three timepoints (baseline and two follow‐up surveys, each separated by 6 months).

Measures used in the cross‐sectional and longitudinal surveys (Study 1 and Study 2) are summarized in Supplementary File 1. Survey questions included items about: (1) the Program, including date ranges of participation in the programme; referral pathways; 11‐point scale‐response perceptions of benefit of, and their satisfaction with, the Program; an open‐ended question inviting comments on the three best things about the Program; and a question about whether they set goals as part of their participation in the Program, and if they did, questions about their experience in setting and progressing towards achieving goals; (2) work demographics regarding employment arrangements for participants in the Program, including their employment and registration statuses, their role and years of clinical experience; (3) personal demographics such as gender, age, ancestry, relationship status, educational level; (4) consent to being contacted for interview; (5) existing validated measures to assess well‐being and ill‐being, as well as associated parameters, in both general and work‐related domains. To assess general well‐being, questions were taken from the Work on Wellbeing (WoW) survey to assess happiness, life satisfaction and health and lifestyle; the full Flourishing Scale (Diener et al., 2010) was also included. To assess work‐related well‐being, questions were taken from the WoW, and the Utrecht Work Engagement Scale (UWES, Schaufeli et al., 2006) and three additional questions requested by the study sponsor (see Supplementary File 1 for further details on additional questions). To assess general ill‐being, questions were taken from the Kessler‐10 (K10, Kessler et al., 2003) and the Stress subscale of the DASS (Lovibond & Lovibond, 1996) were included. To assess work‐related ill‐being, the work‐related burnout scale of the Copenhagen Burnout Inventory (Kristensen et al., 2005) was included. Strengths Use and Strengths Knowledge were also assessed by questions selected from the Strengths Use and Strengths Knowledge scale (Govindji & Linley, 2007).

For Study 3, using a descriptive qualitative study design (Sandelowski, 2010), semi‐structured, in‐depth, approximately 1‐h, interviews were conducted online via zoom between October 2022 and January 2023 (RJ & HB). The interview guide is provided in Supplementary File 1. Interviews were recorded then transcribed using OtterAI™ prior to analysis.

For Study 4, data were collected using a battery of researcher‐developed demographic, Likert scale, rating questions and free response questions. These questions were specifically developed to address the research questions and objectives of this study as no relevant and specific validated instruments were identified in a comprehensive review of the literature.

#### 4.2.2 Sampling and recruitment

A summary of the participants and associated recruitment methods is presented in Supplementary File 2. For studies one and two, programme participants who had indicated they may be contacted for evaluation purposes, and who had been involved with the programme at any point since its inception up until May 2022, were contacted. Potential study participants (*n* = 1464) were invited to the survey via RedCap email with a unique survey link. Participants were informed that upon completion of the survey they would be entered into the draw to win a $50AUD voucher (they could opt out of the draw by checking a box in the survey). Up to two reminder emails were sent. Baseline data were collected from April to July 2022. Of the 1464 contacted, 228 email addresses either no longer worked (bounced) or were incorrect. Of the remaining 1236, a total of 136 (11%) provided consent to participate. Of the 136, a total of 21 answered only the first question thus were excluded, the remaining respondents with sufficient data for analysis were 115. Participants were separated into two cohorts. Of the 115 respondents, 84 engaged with the Programme before 2020 so these participants completed just the cross‐sectional survey (Study 1). The remaining 31 respondents engaged from 2020, so these participants were enrolled in Study 2 (longitudinal survey) and completed the same survey two more times, at 6‐month (October 2022) then 12‐month (April 2023) follow‐up.

For Study 3, participant selection was through prospective sampling of nurses and midwives who had participated in the programme, completed the electronic survey of Study 1 or Study 2, and opted into interview by selecting ‘yes’ to the invitation in a survey item. These programme participants were then contacted by e‐mail and invited to interview (*n* = 51), with 16 providing consent. All current programme clinicians (*n* = 8) and 3 former clinicians were invited by e‐mail; in total 8 provided consent.

For Study 4, key stakeholders were identified through the study sponsor, study adviser and state‐wide academic, health and professional industry publicly available contact e‐mail addresses. In total, 144 stakeholders were invited to participate. The lead researcher contacted stakeholders via email and invited them to participate by following a link to an anonymous online survey. Of the 144 stakeholders, 39 (27%) provided consent. The survey was open for 3 weeks from 8 August to 31 August 2022.

#### 4.2.3 Data analysis

For studies 1 and 2, survey data were exported as .csv files from RedCap, then imported into IBM SPSS Statistics for Windows, version 29 (IBM Corp., Armonk, N.Y., USA) for analysis. Study participants who answered no more than the questions about the dates that they were engaged with the programme were discarded from the analysis; however, partial responses were otherwise analysed and the number of missing responses is reported for each question. For questions about the respondents' role, registration and employment at the time of their participation in the Program, responses of ‘I can't remember’ were recoded in SPSS as a missing value. For each outcome variable at each timepoint, descriptive statistics (mean, standard deviation and range) were calculated. Prior to conducting repeated measures analyses, Q–Q plots were inspected, and the Shapiro–Wilk test was performed for each variable to identify variables that markedly deviated from normality. Given that there was evidence that some variables deviated from normality, the non‐parametric Friedman test was used to determine whether there were changes in any of the outcome variables over time in addressing RQ2, and the Mann–Whitney *U*‐test was used to compare the two cohorts in addressing RQ1.

For Study 3, during and after the reflexive thematic analysis of transcripts researchers (RJ, HB and NB) met, explored and addressed preconceived expectations related to the research, and how these may have influenced development of the research questions, methods, interview questions and analysis (Braun & Clarke, 2022). Two of the researchers were ‘insiders’ as registered nurses, and one a health and social sciences researcher—all three identified as female and had formal research training (Doctor of Philosophy). None of the research team had experience as a participant nor clinician of the programme being evaluated, or had previously engaged with any study participants. Participants were not asked if they were alone or with others at the time of interview. Interprofessional peer review and debriefing supported conceptualization, as recommended by Morse (2015), exploring researchers' positioning and influence on the research. Through the analysis, researchers constructed a description of nurses and midwives experiences of the programme from the perspective of both participants and clinicians adopting the six‐step inductive thematic analysis approach of Braun and Clarke (2022). This approach included (1) familiarizing self with data, (2) generating initial codes, (3) searching for themes, (4) reviewing themes, (5) defining themes and (6) producing the report. Steps 1 to 3 were conducted independently by the researchers using the transcripts, with the support of data management tools NVivo™, Microsoft Word™ or Excel™. Then at Step 4 the initial themes and subthemes were reviewed and refined by the researchers together until gaining consensus and finally naming the themes, where meaning was generated through the interpretation of the data (for elaboration see Braun & Clarke, 2022). Findings were reported as a narrative summary supported by the nurses and midwives' quotes. The themes identified were considered alongside the survey data and then synthesized as part of the overarching programme evaluation.

For Study 4, survey data were exported as .csv files from RedCap, then imported into IBM SPSS Statistics for Windows, version 29 (IBM Corp., Armonk, N.Y., USA) for analysis. For each outcome variable, descriptive statistics (mean, standard deviation and range) were calculated. Free response questions were organized using content analysis (HB and RJ) and arranged in Excel, then frequencies and sample quotes reported.

Data from each of the four studies were analysed and reported separately according to relevant research questions. Where multiple sources of data informed a single research question, these were reported separately, then triangulated and synthesized similar to that of a case study framework (Fetters et al., 2013). Data are finally integrated and presented in a joint display of findings (Fetters & Tajima, 2022).

#### 4.2.4 Ethical approvals

This mixed methods evaluation was approved by the Human Ethics Committee of the University of Melbourne (2022‐22821‐25367‐3). Informed consent was obtained from all participants. For the survey this included integration of the plain language statement and consent form into the RedCap survey. Respondents were informed that their responding to the survey questions constituted consent to participate. Programme participants and clinicians provided written consent prior to interview. Evaluation procedures were conducted according to those approved by the committee to ensure participants and data were protected, and privacy and confidentiality of data maintained.

## 5 RESULTS

Results are reported for each of the four RQs. Where data from multiple studies inform the question, each relevant study is reported independently then synthesized.

### 5.1 RQ1: What are the characteristics of nurses and midwives who engaged in the programme?

The cross‐sectional and longitudinal (T1) surveys contributed to answering RQ1, *What are the characteristics of nurses and midwives who engaged in the programme?* Of the 1236 potential participants, 136 consented to participate, representing a response rate of 11%. Of the 136 people who consented to participate in a survey, 21 completed no more than the first two questions (i.e. regarding the dates that they were engaged with the Program) and were excluded from analysis. Of the remaining 115 participants included in the analysis, 84 reported engaging with the programme prior to January 2020 and therefore completed the cross‐sectional survey, and 31 reported engaging with the programme after 2020 and therefore were provided the opportunity to complete the longitudinal survey. The results presented in this section are based on the responses of the 115 participants who completed either the cross‐sectional survey or the first timepoint of the longitudinal survey.

#### 5.1.1 Demographic profile of nurses/midwives who accessed services of the Program

The personal demographic characteristics of the respondents at the time when they participated in the Program are summarized in Table 1.

**TABLE 1:** Participant characteristics.

Variable	Programme participant prior to 2020 (*n* = 84)		Programme participant in 2020 or later (*n* = 31)		Total (*n* = 115)	
	*N*	%	*N*	%	*N*	%
Gender						
Female	59	87	26	93	85	89
Male	9	13	2	7	11	11
Missing	16	‐	3	‐	19	‐
Children						
Yes	45	67	19	68	64	67
No	22	33	9	32	31	33
Missing	17	‐	3	‐	20	‐
Relationship status						
Single	15	22	5	18	20	21
In a relationship	11	16	5	18	16	17
Married	30	44	15	54	45	47
Divorced or separated	9	13	3	11	12	13
Other or prefer not to say	3	4	0	0	3	3
Missing	16	‐	3	‐	19	‐
Highest level of education completed						
College/university graduate/TAFE	23	34	11	39	34	35
Postgraduate certificate	14	21	6	21	20	21
Postgraduate diploma	18	27	9	32	27	28
Masters' degree	9	13	0	0	9	9
Professional doctorate or PhD	1	2	0	0	1	1
High school certificate	3	4	2	7	5	5
Missing	16	‐	3	‐	19	‐
Ancestry^f^						
Oceanian	16	24	5	18	21	22
North‐west European	31	46	15	54	46	48
Southern and Eastern European	6	9	2	7	8	8
North African/Middle Eastern/sub‐Saharan African	1	1	0	0	1	1
Asian (south‐east or north‐east or southern and central)	2	3	3	11	5	5
Prefer not to say	12	18	3	11	15	16
Missing	16	‐	3	‐	19	‐
Aboriginal or Torres Strait Islander						
Aboriginal	0	0	1	4	1	1
Torres Strait Islander	0	0	0	0	0	0
Neither Aboriginal nor Torres Strait Islander	68	100	26	93	94	98
Prefer not to say	0	0	1	4	1	1
Missing	16	‐	3	‐	19	‐
Employment status as participant						
Employed but not working	10	13	10	33	20	19
Unemployed	7	9	2	7	9	9
Working <35 h per week	31	41	9	30	40	38
Working >35 h per week	25	33	9	30	34	32
Cannot remember	3	4	0	0	3	3
Missing	8	‐	1	‐	9	‐
Registration as participant						
RN	49^a^	64	23	77	72^d^	68
RN1	2	4	2	9	4	6
RN2	8	16	6	26	14	19
RN3	5	10	1	4	6	8
RN4	1	2	1	4	2	3
RN5	2	4	0	0	2	3
RN6	1	2	0	0	1	1
RN7	4	8	1		5	7
Clinical nurse specialist	8	16	5	21	13	18
ANUM/NUM	10	20	4	17	14	19
Clinical nurse consultant	3	6	0	0	3	4
Educator	3	6	2	9	5	7
DoN/Adon	1	2	0	0	1	1
Other	1	2	1	4	2	2
RM	10^b^	13	3^c^	10	13^e^	12
RM1	1	10	0	0	1	8
RM2	2	20	0	0	2	15
RM3	1	10	1	33	2	15
RM4	1	10	0	0	1	8
RM5	1	10	0	0	1	8
RM7	1	10	0	0	1	8
Clinical midwife specialist	2	20	1	33	3	23.1
AMUM	1	10	0	0	1	8
Other	0	0	1	33	1	8
EN	9	12	3	10	12	11
Graduate RN (first year of practice)	3	4	1	3	4	4
Graduate RM (first year of practice)	1	1	0	0	1	1
Student RN	2	3	0	0	2	2
Student EN	1	1	0	0	1	1
Cannot remember	1	1	0	0	1	1
Missing	8	‐	1	‐	9	‐
Age at survey completion						
Mean (SD)	52.14 (12.21)		47.96 (9.80)		50.89 (11.65)	
Range	26–71		28–64		26–71	
Missing	18		3		21	
Years of clinical experience as a participant						
Mean (SD)	18.85 (14.23)		21.07 (11.15)		19.48 (13.41)	
Range	0–47		1–40		0–47	
Missing	11		2		13	

Abbreviations: ADOM, associate director of midwifery; ADON, associate director of nursing; AMUM, associate midwifery unit manager; ANUM, associate nurse unit manager; DoM, director of midwifery; DoN, director of nursing; EN, enrolled nurse; MUM, midwifery unit manager; NUM, nurse unit manager; RM, registered midwife; RN, registered nurse; TAFE, technical and further education.

^a^ Including 1 who reported being dual registered RN/RM but worked predominantly as an RN.

^b^ Including 9 who reported being dual registered RN/RM but worked predominantly as an RM.

^c^ Including 3 who reported being dual registered but worked predominantly as an RM.

^d^ Including 1 who was dual registered.

^e^ Including 12 who were dual registered; percentages are calculated excluding the number of missing values from the denominator and are rounded to nearest whole number, therefore, may not sum to exactly 100.

^f^ No respondents selected ‘Peoples of the Americas’ as ancestry.

The average age at which the nurses/midwives accessed the Program was 51, and most participants identified as females (89%) who were married (47%) with children (67%). One respondent (in the post‐2020 cohort) reported identifying as aboriginal.

Most study participants reported being employed at the time of accessing the Program; less than 10% reported having been unemployed at the time of participating in the programme. Respondents were primarily RNs (68%), with fewer being registered as an EN (11%). Very few (less than 5%) respondents reported being a graduate (first year of practice) or student at the time of participating in the Program. Overall, Program participants had a wide range of years of clinical experience (0–47 years), with an average of 19 years. This distribution of results demonstrates that nurses/midwives who access the programme were spread across a wide range of professional classification levels.

#### 5.1.2 Well‐being and ill‐being profiles of nurses/midwives who accessed services of the Program

A descriptive summary of levels of well‐being and ill‐being, both in general and work‐related domains for both cohorts are provided in Supplementary File 3. Associations between well‐being and ill‐being variables in general and work‐related domains, are summarized descriptively in Table 2.

**TABLE 2:** Associations between variables for well‐being and ill‐being.

	1.	2.	3.	4.	5.	6.	7.	8.	9.	10.	11.	12.	13.	14.	15.	16.	17.	18.	19.	20.	21.	22.	23.
General well‐being																							
1. Health	1.00																						
2. Flourishing	.598**	1.00																					
3. Happiness	.582**	.531**	1.00																				
4. Life satisfaction	.656**	.733**	.779**	1.00																			
Work well‐being																							
5. Job Satisfaction	.428**	.408**	.359**	.471**	1.00																		
6. Work–life balance	.456**	.332**	.329**	.387**	.699**	1.00																	
7. Work happiness	.527**	.510**	.434**	.579**	.911**	.699**	1.00																
8. Work relationships	.422**	.460**	.398**	.497**	.734**	.625**	.757**	1.00															
9. Work vitality	.524**	.441**	.449**	.556**	.882**	.648**	.891**	.704**	1.00														
10. Work motivation	.499**	.408**	.384**	.493**	.787**	.546**	.786**	.587**	.862**	1.00													
11. Valued by manager	.350**	.326**	.277**	.377**	.799**	.570**	.756**	.701**	.711**	.673**	1.00												
12. Valued by organisation	.325**	.323**	.220*	.256*	.758**	.521**	.706**	.578**	.656**	.659**	.797**	1.00											
13. My work makes a difference	.405**	.447**	.310**	.427**	.630**	.375**	.616**	.525**	.648**	.707**	.514**	.521**	1.00										
14. Ability to disconnect after work	−.12	.03	−.04	.03	−.14	−.199*	−.07	−.06	−.08	−.09	−.15	−.05	−.01	1.00									
15. Work pride	.374**	.460**	.263**	.397**	.522**	.277**	.521**	.308**	.536**	.616**	.408**	.416**	.712**	−.01	1.00								
Work ill‐being																							
16. Work inspiration	.422**	.510**	.330**	.447**	.674**	.362**	.669**	.438**	.710**	.770**	.530**	.541**	.758**	−.02	.789**	1.00							
17. work stress	−.09	.12	.07	.14	−.10	−.199*	−.06	−.02	−.03	−.06	−.13	−.13	.15	.486**	.06	.05	1.00						
18. Burnout	−.350**	−.210*	−.299**	−.205*	−.589**	−.459**	−.565**	−.420**	−.550**	−.479**	−.592**	−.482**	−.281**	.428**	−.255*	−.362**	.495**	1.00					
General ill‐being																							
19. DASS	−.18	−.286**	−.447**	−.298**	−.244*	−.12	−.301**	−.261**	−.297**	−.283**	−.313**	−.16	−.19	.323**	−.19	−.269**	.14	.454**	1.00				
20. Kessler‐10	−.304**	−.375**	−.443**	−.357**	−.315**	−.275**	−.361**	−.300**	−.361**	−.304**	−.319**	−.19	−.246*	.223*	−.230*	−.335**	.246*	.417**	.694**	1.00			
21. Brief Resilience Scale	.401**	.300**	.538**	.438**	.08	.09	.205*	.09	.251*	.213*	.10	.00	.20	−.19	.19	.17	.04	−.203*	−.504**	−.450**	1.00		
22. Strengths knowledge	.297**	.336**	.239*	.321**	.228*	.263*	.222*	.252*	.250*	.209*	.226*	.252*	.346**	−.12	.358**	.17	.03	−.12	.01	−.04	.217*	1.00	
23. Strengths use	.430**	.479**	.393**	.549**	.443**	.400**	.479**	.427**	.476**	.467**	.424**	.387**	.445**	−.14	.415**	.342**	−.06	−.326**	−.11	−.15	.272**	.819**	1.00
Mean	58.42	44.1	6.36	6.76	6.01	5.94	5.83	2.28	5.80	6.57	5.73	4.54	7.31	5.04	4.75	4.06	6.85	50.7143	17.7	20.19	31.36	23.44	20.71
*SD*	19.039	9.18	2.11	2.01	2.71	2.64	2.74	2.80	2.72	2.68	3.29	3.14	2.55	2.81	1.16	1.45	2.49	22.58199	10.27	8.80	9.19	5.71	6.06
Range	0–100	8–56	1–9	1–10	0–10	0–10	0–10	0–10	0–10	0–10	0–10	0–10	0–10	0–10	0–6	0–6	0–10	3.75–100	0–42	10–50	1–48	0–30	0–30
*N*	103	103	105	104	100	100	98	99	100	100	99	99	99	100	100	100	100	100	100	98	103	97	97

*Note*: Correlations greater than .4 are highlighted.

Abbreviations: DASS, Depression, Anxiety and Stress Scale (Stress subscale only); K10, Kessler 10‐item scale.

* Correlation is significant at the 0.05 level (2‐tailed).

** Correlation is significant at the 0.01 level (2‐tailed).

#### 5.1.3 Work well‐being and ill‐being

Overall, the pattern of results indicates low levels of work well‐being, with the mean scores of individual items approximately at the midpoint of the scale range for most items. The work well‐being domain most favourably endorsed was the perception that their work ‘makes a difference’. The positively worded work well‐being domain rated least favourably was the perception that they were ‘valued by the organization’ that they work for. The negatively worded work well‐being domain, perceived work stress, was rated poorly by most respondents. For all domains except the perceived ability to disconnect from work, respondents who participated in the Program prior to 2020 reported greater well‐being than those who participated since 2020. Burnout levels reported in the present study were greater than those reported in a study of Australian midwives (Creedy et al., 2017).

For each domain, Mann–Whitney tests were carried out to compare the work well‐being of the pre‐2020 and post‐2020 cohort; statistically significant differences were present for 2 of these 11 work well‐being domains: job satisfaction (*U* = 749.00, *p* = .032); and work stress (*U* = 725.00, *p* = .019). In terms of burnout, the group mean for the pre‐2020 cohort (*M* = 47.12) fell within the ‘Low/No Burnout’ range, and the group mean for the post‐2020 cohort (*M* = 59.95) fell within the ‘Moderate Burnout’ range; there was a statistically significant difference between the two groups (*U* = 713.50, *p* = .024). In summary, lower levels of work well‐being were reported by respondents who participated in the Program since 2020 in comparison with those prior to 2020.

#### 5.1.4 General well‐being and ill‐being

Well‐being was assessed with several variables, including the Flourishing Scale, the composite indicator of satisfaction with health and lifestyle based on four items from the WoW questionnaire, a question about happiness, and a question about life satisfaction. There were no statistically significant differences between the two cohorts on any of the general well‐being variables. Notably, the level of flourishing was lower than that reported by a group of nurses surveyed in Victoria during the 2020 wave of the pandemic (Jarden et al., 2022).

Ill‐being was assessed with two scales: the Stress scale of the DASS and the K‐10. There were no statistically significant differences between the two groups for either indicator. Notably, stress levels were higher than those reported by a sample of nurses during the 2020 wave of the pandemic in Victoria (Delgado et al., 2021). According to the clinically relevant categories of the DASS Stress scale, the group average (*M* = 17.72) was in the mild category. The group mean (*M* = 20.19) for the K10 was in the mild distress category and was not significantly different to a group of 433 hospital workers in New South Wales in 2020 (Stubbs et al., 2021).

#### 5.1.5 Domains that influence well‐being and ill‐being

Three variables that have been identified as relating to well‐being and ill‐being—resilience, strengths use and strengths knowledge—were also assessed. There were no statistically significant differences between the cohorts for these three variables. Notably, the level of resilience in the present study was lower than that reported by a sample of Victorian nurses during the COVID‐19 pandemic in 2020 (Jarden et al., 2022).

#### 5.1.6 Associations between study variables

To explore associations between the study variables Pearson correlation coefficients were explored. Moderate‐to‐strong correlations (*r* ≥ |.4|) were highlighted. Health and lifestyle indicators had a moderate correlation with other well‐being indicators, and with most work well‐being indicators. The health and lifestyle score was also correlated with resilience and with strengths use (although not strengths knowledge). None of the satisfaction with Program variables correlated with well‐being or ill‐being indicators. Resilience was not associated with any of the work well‐being variables or burnout. Resilience was inversely associated with stress (as measured by the DASS‐Stress Subscale, *r* = −.504) and inversely associated with psychological distress (as measured by the Kessler‐10, *r* = −.450). Resilience was positively associated with happiness (*r* = .538) and life satisfaction (*r* = .438). Correlations were also explored between all variables and age at the time of participating in the Program, and years of clinical experience when engaging with the Program; none of the study variables were correlated with age or clinical experience (not shown).

### 5.2 RQ2: What is the effectiveness of the case management model on the well‐being of nurses and midwives?

The longitudinal survey (study 2) contributed to answering RQ2 *What is the effectiveness of the case management model on the well‐being of nurses and midwives?* Of the 31 respondents from the baseline survey who reported engaging with the programme after 2020, 22 went on to consent to the mid‐point survey (6‐month follow‐up), and 16 went on to consent to the end‐point survey (12‐month follow‐up). Fifteen respondents completed surveys at all three timepoints. At the second timepoint (6‐month follow‐up), 8 of 22 respondents (36%) reported having accessed the services of the Program in the time since having completed the baseline survey. At the third timepoint (12‐month follow‐up), 9 of 16 respondents (56%) reported having accessed services of the Program since completing the previous survey. Therefore, at the final timepoint, approximately half of the sample had engaged with the Program at some point during the 6 months preceding April 2023, and approximately 50% of the respondents had not engaged with the programme for at least 6 months.

A descriptive summary of levels of well‐being and ill‐being, both in general and work‐related domains are summarized descriptively in Table 3.

**TABLE 3:** Summary of well‐being and ill‐being indicators, across three timepoints.

Variable	Baseline				Midpoint				Endpoint				Friedman test significance
	Mean (Std. deviation)	Min.	Max.	*N*	Mean (Std. deviation)	Min.	Max.	*N*	Mean (Std. deviation)	Min	Max.	*N*	
Work well‐being													
Job satisfaction	5.10 (2.81)	0.00	10.00	29.00	5.95 (2.60)	0.00	9.00	21.00	4.31 (3.16)	0.00	8.00	16.00	*χ* ^2^(2), 8.35, *p*, .015
Work life balance	5.45 (2.95)	0.00	10.00	29.00	5.62 (2.60)	0.00	9.00	21.00	4.50 (2.78)	0.00	10.00	16.00	*χ* ^2^(2), .76, *p*, .684.
Work happiness	5.11 (2.78)	0.00	8.00	28.00	5.81 (2.82)	0.00	9.00	21.00	4.69 (3.05)	0.00	9.00	16.00	*χ* ^2^(2), 5.66, *p*, .059
Work relationships	5.97 (3.01)	0.00	10.00	29.00	6.48 (2.73)	0.00	10.00	21.00	5.13 (3.63)	0.00	10.00	16.00	*χ* ^2^(2) = .522, *p*, .770
Work vitality	4.93 (2.91)	0.00	10.00	29.00	5.29 (3.07)	0.00	9.00	21.00	4.81 (3.29)	0.00	10.00	16.00	*χ* ^2^(2) =3.5, *p* = .174
Work motivation	5.72 (2.85)	1.00	10.00	29.00	5.81 (3.04)	0.00	10.00	21.00	5.13 (3.01)	0.00	9.00	16.00	*χ* ^2^(2) = .565, *p*, .754
Valued by manager	5.10 (3.48)	0.00	10.00	29.00	5.00 (3.58)	0.00	10.00	21.00	4.50 (4.10)	0.00	10.00	16.00	*χ* ^2^(2) = 2.0, *p*, .368
Work stress	7.76 (2.03)	4.00	10.00	29.00	7.05 (2.22)	3.00	10.00	21.00	7.44 (2.53)	2.00	10.00	16.00	*χ* ^2^(2) = .00, *p*, 1.0
Valued by organization	3.83 (3.35)	0.00	10.00	29.00	4.29 (3.36)	0.00	9.00	21.00	3.50 (3.12)	0.00	9.00	16.00	*χ* ^2^(2) = 1.38, *p*, .50
My work makes a difference	7.17 (2.59)	1.00	10.00	29.00	6.76 (2.68)	0.00	10.00	21.00	6.94 (2.59)	0.00	10.00	16.00	*χ* ^2^(2), .326, *p*, .850
Ability to disconnect	5.66 (2.98)	0.00	10.00	29.00	5.62 (2.58)	1.00	10.00	21.00	6.19 (3.39)	0.00	10.00	16.00	*χ* ^2^(2), 1.773, *p*, .412
Work pride	4.54 (1.14)	2.00	6.00	28.00	4.62 (1.24)	2.00	6.00	21.00	4.44 (1.31)	2.00	6.00	16.00	*χ* ^2^(2), .071, *p*, .965
Work inspiration	3.83 (1.42)	1.00	6.00	29.00	3.85 (1.60)	0.00	6.00	20.00	3.38 (1.78)	0.00	6.00	16.00	*χ* ^2^(2), 2.46, *p*, .292
Work ill‐being													
Burnout—work subscale	59.95 (21.79)	29.00	100.00	28.00	57.65 (21.38)	17.86	92.86	21.00	64.06 (19.54)	25.00	92.86	16.00	*χ* ^2^(2), 1.107, *p*, .575
General well‐being													
Flourishing Scale (8–56)	44.00 (7.47)	17.00	53.00	29.00	44.32 (5.92)	33.00	55.00	22.00	44.56 (5.24)	31.00	51.00	16.00	*χ* ^2^(2) = .448, *p*, .799
Health and lifestyle	57.75 (14.39)	25	92.5	30	53.75 (15.62)	17.5	82.50	22	54.69 (14.05)	22.5	77.5	16	*χ* ^2^(2) = .691, *p*, .708
Happiness	6.27 (2.21)	1	8	30	6.14 (1.08)	3	10	22	6.12 (1.59)	2	8	16	*χ* ^2^(2) =1.773, *p*, .412
Life satisfaction	6.67 (1.94)	3	10	30	6.55 (1.87)	2	10	22	6.44 (1.09)	5	8	16	*χ* ^2^(2), .792, *p*, .673
Resilience	31.45 (11.00)	4.00	48.00	29.00	29.33 (10.71)	3.00	51.00	21.00	28.00 (8.90)	2.00	41.00	16.00	*χ* ^2^(2), .943, *p*, .624
General ill‐being													
DASS—stress	20.5 (10.09)	4.00	42.00	28.00	20.48 (7.61)	8.00	38.00	21.00	20.5 (8.90)	6.00	36.00	16.00	*χ* ^2^(2), .778, *p*, .678
K‐10	20.64 (6.67)	12.00	33.00	28.00	22.14 (5.76)	15.00	33.00	21.00	20.33 (5.41)	13.00	33.00	15.00	*χ* ^2^(2) =2.92, *p*, .232
Strengths knowledge	23.00 (5.44)	9.00	30.00	28.00	23.24 (3.87)	17.00	30.00	21.00	23.00 (4.23)	17.00	30.00	15.00	*χ* ^2^(2) = .00, *p* = 1.00
Strengths use	19.46 (5.87)	6.00	30.00	28.00	18.33 (6.70)	2.00	30.00	21.00	17.67 (7.23)	1.00	29.00	15.00	*χ* ^2^(2) = .264, *p*, .876

Abbreviations: DASS, Depression, Anxiety and Stress Scale (Stress subscale only); K10, Kessler 10‐item scale.

Regarding work well‐being, there was no statistically significant changes across any of the work well‐being variables over time, except for Job Satisfaction, which declined from baseline to timepoint three (12‐month follow‐up). Burnout increased over time from a mean of 60 (*SD* 22, range: 29–100) to 64 (SD = 20, range: 25–93); however, this change was not statistically significant. Over time, none of the general well‐being indicators changed substantially. As indicated by the comparator sample, the respondents in the present study scored lower on the Flourishing Scale than a sample of 49 nurses during the 2020 pandemic in Victoria (Jarden et al., 2022). Over time, levels of stress (as measured by the DASS Stress subscale) and psychological distress (as measured by the K‐10) were relatively stable. There were no significant changes over time for strengths use, strengths knowledge or resilience. In summary, the longitudinal survey (Study 2) contributed to answering RQ2 *What is the effectiveness of the case management model on the well‐being of nurses and midwives*? There was no change across any of the outcome measures across the three timepoints.

### 5.3 RQ3: What are the experiences and perceptions of nurses and midwives engaging in the programme as participants and clinicians?

The cross‐sectional, longitudinal and qualitative descriptive studies contributed to answering RQ3, *What are the experiences and perceptions of nurses and midwives engaging in the programme as participants and clinicians?* Data from the cross‐sectional (Study 1), longitudinal (Study 2) and qualitative descriptive (Study 3) studies were analysed and reported in the findings independently, then triangulated and contextualized in the discussion. For the cross‐sectional and longitudinal studies, items related to satisfaction with programme, perceptions of the best things about the programme, referral pathways and goal setting were analysed and reported as descriptives (tabulated). Correlations were explored between well‐being and ill‐being variables and participant satisfaction, then described as a narrative summary. For the interviews, transcripts were analysed and are reported thematically. First, analysis of the survey data (cross‐sectional study 1 and longitudinal study 2) was reported for items in which programme participants were asked about satisfaction with the programme, perceptions of the best things about the programme, referral pathways, goal setting, the analysis of correlations between well‐being and ill‐being variables and participant satisfaction. Second, interview analysis of both programme participants and clinicians was reported.

#### 5.3.1 Analysis of survey data

Four items from the cross‐sectional (Study 1) and longitudinal (Study 2) surveys were analysed and reported: (1) satisfaction with the Program, (2) perceptions of the best things about the Program, (3) referral pathways to and from the Program and (4) goal setting as part of the Program. Respondents who had previously engaged with Program answered eight survey questions about their satisfaction with the Program. Satisfaction was high in all areas. Notably, respondents who had engaged with the Program more recently (since 2020) reported greater satisfaction that those who had engaged with the programme before 2020, and this difference was statistically significant for all items (see Supplementary File 4 for full descriptive statistics for each item for each group). It is unclear whether this difference is due to changes in service delivery over time which truly effect participant satisfaction, or whether these results are due to recall bias (Brusco & Watts, 2015; OECD, 2013), which is a well‐established phenomenon whereby the greater the interval between using a service and being surveyed or interviewed about satisfaction with it, the poorer the recall. Associations between the eight participant satisfaction variables and the general and work‐related well‐being and ill‐being variables were also explored with Pearson correlation coefficients (the full correlation matrix is presented in Supplementary File 5). None of these variables were associated with each other.

One hundred and four respondents reported one or more of the ‘best things’ about the Program. After excluding one comment that was not in English, and exclusion of two negative comments, content analysis was performed on 101 comments. A summary of the content and frequency of responses is summarized in Supplementary File 6. The most commonly reported best things were ‘being listened to, being heard, being understood, being validated (*f* = 69), ‘support and help’ (*f* = 35) and ‘by nurses and midwives, for nurses and midwives’ (*f* = 34). Being listened to, being heard, being understood, being validated for one participant included ‘*Understanding. My first consultant was a mental health nurse. She fully understood my situation*’. Support and help for one participant included ‘*Excellent advice and support*’. By nurses and midwives, for nurses and midwives for one participant included ‘*The counsellor had a nursing background herself so she was able to really understand the context unlike other counsellors*’, and for another participant ‘*The opportunity to discuss workplace issues with people who have shared the lived experience of health professionals*’.

Respondents were asked to indicate how they had been referred to the Program; most (62%, *n* = 72) were self‐referred, with fewer reporting that they were referred by work (10%, *n* = 11). Around a fifth of respondents (22%, *n* = 25) reported being referred from other sources, including the union, General Practitioners, psychologists, psychiatrists, other support services. Approximately half of respondents reported accessing other services as a result of participating in the Program, with the most common service being that of a psychologist. A summary of all reported referral pathways to and from the Program are tabulated in Supplementary File 7.

Respondents were asked about goal setting in terms of achievability and progress towards these goals, as part of their experience of the programme (tabulated in Supplementary File 8). Almost half of respondents (46%) indicated that they had set goals when participating in the Program, and on average, perceived that these goals were achievable. These findings regarding the importance of goal setting align with the Program's strategic objectives, which emphasized the principle of person‐centeredness in the model of care.

#### 5.3.2 Analysis of interview data

**FIGURE 2:**
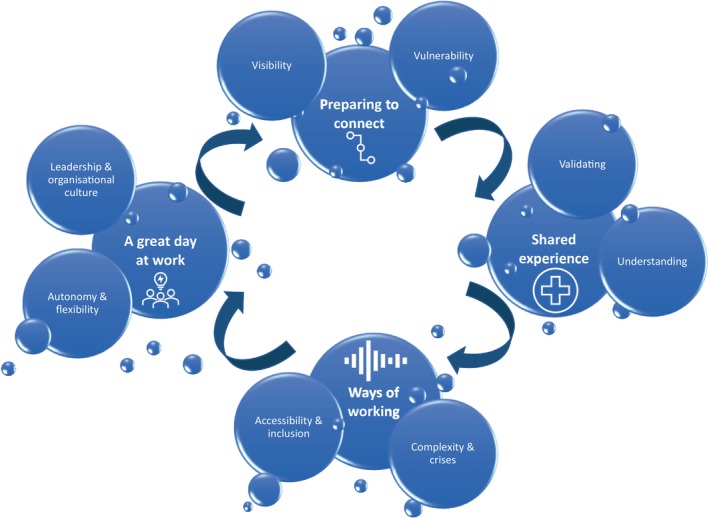
Program participant and clinicians' experiences and perceptions.

Eight clinicians and 16 programme participants were interviewed (HB & RJ) from between 45 min and 2.5 h. For the clinicians, given the small and potentially uniquely identifiable population, demographic data are not reported. For the programme participants, most were nurses with a median age of 54 years (*M* = 51, *SD* 11) who had engaged with the programme between 2011 and 2022 for reasons relating to mental health/well‐being. Thirteen were still practising nurses or midwives. In the analysis of the interview transcripts four themes were constructed encompassing programme participants' and/or clinicians' experiences (see Figure 2).

These four themes included ‘Preparing to connect’, ‘Shared experience’, ‘Ways of working’ and ‘A great day at work’. Theme one, Preparing to connect, encompassed participants experiences, thoughts, and feelings in their steps towards engaging with the programme. Two sub‐themes were constructed, ‘Visibility’ and ‘Vulnerability’. Participants emphasized there should be promotion of the programme widely to support improved visibility and reach, highlighting the value of resources to support prevention and self‐assessment of risk. Ongoing promotion was suggested to reinforce availability of the service and address pre‐conceived barriers to accessing support.

Theme two, Shared experience, encompassed participants' and clinicians' thoughts and feelings about the programme's model of clinicians having lived experience of nursing and/or midwifery practice. Two sub‐themes were constructed, ‘Understanding’ and ‘Validating’. Participants expressed feeling understood and validated through engaging with the Program. This understanding and validation seemed inextricably linked to Program clinicians having lived experience of nursing and/or midwifery practice, a key feature of the Program model of care.

Theme three, Ways of working, encompassed participants' and clinicians' thoughts and feelings of engaging and experiencing the programme and case management model. Two sub‐themes were constructed, ‘Accessibility and inclusion’ and ‘Complexity and crises’. Participants referred to factors that enabled or inhibited their participation, ranging from practical factors to cultural and psychosocial factors. Both clinicians and participants highlighted person‐centric features of the Program that increase its accessibility and adaptability to the individual, including varied meeting formats, low barriers to entry in terms of cost and scheduling, and flexibility around the number of sessions.

Theme four, A great day at work, was constructed as a theme from the experiences, thoughts and feelings of clinicians being prepared for and engaging with programme stakeholders, team and participants. Two sub‐themes were constructed, ‘Leadership and organizational culture’ and ‘Autonomy and flexibility’. Overall, clinicians expressed positive perceptions of work culture at the Program, emphasizing strong leadership, which had flow on effects such as enabling strong and productive teamwork and relationships. Themes, sub‐themes and representative quotes are reported in Table 4.

**TABLE 4:** Themes, sub‐themes and representative quotes.

Theme	Sub‐theme	Theme descriptors and representative quotes
Theme 1: Preparing to connect	Subtheme 1.1: Visibility	At an organizational and operational level, promotion and reach of the programme were felt by clinicians to be a high priority from entry to practice programmes through to graduates and early career nurses and midwives and beyond, focusing on the uniqueness of the programme as a space for nurses and midwives and ensuring all nurses and midwives learn about the programme when they are well, or can easily find it when they need it. Drawing from technology and social media, champions, nursing and midwifery leadership and management and collective strengths of stakeholders were all suggested mechanisms for promotion, ‘*our social networking, all of our promotional stuff, like when you look at other programmes, they have whole teams that do that. And it's always been left to us, so it's always very* ad hoc*, and like, we're flying by the seat of our pants all the time trying to make that happen…nurses and midwives are so incredibly busy that they only remember what they need at that time. Yeah. And so if they don't need it, then and then it's forgotten. So they might have heard of us somewhere along the line. But if they don't need us, they won't remember. Yeah. And hence the marketing and social media, what it like whatever marketing, you know, like, if we had a whole department that was doing that, potentially everyone would know about us*’. (clinician) Participants initially found the programme through a range of avenues such as a website, brochure, poster, through to referrals from union or a colleague, hearing of others positive experiences and the champions programme. More promotion and stronger local referral systems, such as by health organization senior management, were proposed by participants to be important, ‘*I would love to see if it's facilitated, if it can be facilitated, or I would love to see, some in‐service on a local level, particularly, like I said, […] we're dealing with a lot of ‐ this is the perfect opportunity and the perfect time for the Program to get in and get involved. […] there's a lot of let's just say, you know, we're not exactly leading with kindness at the moment. So I think this is the perfect opportunity for Program to get involved because we've just come off the back the back end of a two‐year lockdown. People's mental health is not great, people's drug and alcohol problem ‐ people's drug and alcohol misuse and substance abuse rates are up. And I think I would love to see on a local level, Program sort of stepping in and being like: We're here, it's confidential, we're a service that can provide support for whatever you're going through*’. (RN371) Promotion was suggested as a means to indirectly save patient lives, underpinned by early intervention and prevention, ‘*Think of the prevention, think of how many nurses you hear the stories of like they're struggling, and everyone knows that they're two that they're one bad day away from killing someone? Yeah. What would happen if there were if they had been referred to the service that I had been? Where, how many people could be saved? If there was someone there saying, hey, there is a service here. Use it. Figure out, get yourself together. Because you're about to kill someone. It will save lives. It probably already has*’ (EN81) ‘*There was a roundtable setting talking a bit about mental health. One of the best things there […] was a really good slide that I actually wanted to get a copy of about assessing whether you're cruising – ‘Okay’, you know, slightly at risk or highly at risk […] which I thought was really valuable in terms of identifying, you know, whether your your mental health is at risk because of what's going on. So I found that really useful*’. (RN/RM224)
	Sub‐theme 1.2: Vulnerability	There was an acknowledgement of more recent promotion local in the organizations by industry (programme) experts and through social media. This promotion was felt to be particularly important to break down ‘taboo’ and clarify misunderstandings for those who might not be sure if the fit the ‘category’ to contact the programme, ‘*So even though it wasn't a nursing‐related issue, because I was worried that, you know, wasn't nursing related. But because I was still actively working and needed to perform my role as a nurse with my background, the background problem going on, and to so the best things about the first phone call, yeah, for me was relief, that I wasn't dismissed as not worthy of the programme. I suppose that was probably the biggest relief that I was actually listened to*’. (RN1170) Additionally, there was *fear in making contact*, where nurses and midwives expressed feeling worried, nervous, apprehensive and vulnerable on engagement, ‘*…just all those just all those normal those normal gendered things that you know, that you possibly like a sign of weakness talking about these things, and sort of like, you know, making like, you know, making like too much out of it and all that sort of thing and possibly, possibly, yeah, possibly coming across. It's all that it's all that idea about sort of, like, you know, sharing, you know, sharing sort of, you know, relatively private things about the workplace with people that you don't really know. So possibly feeling possibly feeling vulnerable and exposed*’ (RN51) The opportunity to speak with someone unrelated to their employer's organization appealed to several who were looking for a confidential service independent from their place of employment, ‘*the hospital does have the counselling, but I went to see one person one time and just their language and the how they were putting it was like that, yeah, this this is not going to work. And I think I tend to be very distrustful of management where I work and so even just knowing it's their programme even though I know rationally that it's confidential and you know that it's what's the word I'm looking for. Impartial. I think I had sort of resentments and things there. So I prefer to seek it out in other avenues*’ (EN31)
Theme 2: Shared experience	Sub‐theme 2.1: Understanding	Clinicians' felt their lived experience of nursing and/or midwifery practice was invaluable, alongside the broad diversity in clinician experiences to support the delivery of the programme, ‘*You know, you've got that background knowledge, you know, all the intricacies that other people don't understand in terms of, you know, the lingo, the language, the, you know, the experience of, you know, the demands of working in a busy ward, you know, that connection sort of on that level*’. (clinician) Participants resoundingly expressed the importance of the clinicians being colleagues or peers, and sharing their lived experience of nursing and/or midwifery, ‘*I was able to give real examples of in, in my workplace, and the way I was feeling and my behaviours and what I was doing and what was concerning, we kind of went through scenarios, I could explain scenarios to them. And they understood because they'd been in a similar workplace. Yeah*’. (RNRM 492) Seeking support from a colleague, a peer, someone who understood, was meaningful to participants, particularly for those who had spoken with other professionals who did not have the foundation of a shared understanding, ‘*for a number of other issues, that people might go to the [programme] with, I think those things would be very much helped by going to that programme, partly because, you know, it's, it's pretty much run by nurses, for nurses. And I think that's an incredible strength. […] if you haven't been a nurse, you can't really understand what it's like to be a nurse. And that's why that's what I think is a real strength of the programme. And I think that's what sets it apart from [other Programmes]… being able to speak to somebody who knows what a late early is for is, you know, is a really good is a really good foundation, because it's hard, I think, for nurses to ring up and say, I need some I need support. I'm not, I'm not okay. And to be able to ring somebody who has kind of stood in your shoes is, I think, the essence of its success, really, because you know, that's the thing that bonds you. Understanding*’. (RN453) The shared understanding, having someone knowledgeable of their work and speaking with someone with a shared professional language was invaluable to participants. This shared understanding felt reassuring and enabled vulnerability, a sense they did not need to translate as they were understood, ‘*it's easier, because you don't translate … so you can start off, the conversation can flow. And you're getting to the nitty gritty of things…Just inherently, it's so much simpler. And it just takes one more level of the dialogue out, because you can just cut to the chase and they equally can respond with something that you can understand because it's how, our language is different, I guess. Yes. Because of the nature of it, our jobs and caring, and empathy, and all those things. So you've got this professional, clinical critical thinking kind of role, but wrapped up in emotion. So you've got really good at balancing those two things and peeling them apart for you. So you can see that one might be compromising the other or having an effect on the other. And it's really, I found helpful*’. (RN308) Participants considered it important that this was a programme of nurses and midwives supporting each other, ‘*there's been a lot said about, nurses, you know, they eat their own young and, and things like that. It can be true to some respect, some nurses don't support each other. And I think with that programme, you've got nurses supporting nurses*’. (EN594)
	Sub‐theme 2.2: Validating	Participants overwhelmingly spoke of the programme being fantastic, exceeding expectations, that it was essential, and shared their positive feelings about the programme, ‘*…I just can't talk about it highly enough. I speak about it quite openly. I'm not ashamed to tell people that I've used it. You know, when I've had things going on, and, you know, I think that that whole thing of just acknowledging, yeah, what you're going through is real, it has an impact, not just on your mind, but your body as well*’. (RN/RM224) ‘*I just hope that the people who run the programme know that, you know, I had a happy ending*’. (EN81) ‘*I would love to go back and see [clinician name]. And just for a success story for her I think and just to just to say that she can pass that on*’. (RN371) These positive statements extended to their experiences working with the clinicians relating that they felt validated, comfortable, supported, understood, nurtured and listened to, ‘*I felt that she understood. Yeah. So maybe it was just the right person the right time. I think that makes a lot of difference […] and that was probably the best thing I ever did. Just finally had someone to talk to independently about it*’. (RN294) ‘*I found it inclusive, I found they were very sort of, you know, open to my problems that they you know, weren't judgmental, they made me feel like, you know, what I was saying…They made me feel like my, you know, like my concerns my issues mattered. Like they that they weren't kind of dismissed as something as something trivial and just you know, go back and back up and you'll be okay. So I was taken seriously Yes, I was taken seriously*’. (RN51) ‘*I felt, you know, I guess relieved in myself that I was doing something about my problem, and that I was taking a step to, I was acknowledging that I guess I needed help and acknowledging that there was a problem and acknowledging that it was okay to ask for help. So I think I felt relief from that sense that I was now acknowledging a problem and getting help with it*’. (RN371) Participants also reported that knowing that they were not alone was important, ‘*[hearing of others] who had gone through what I had gone through and had come out the other side and had been successful […] So listening to those sorts of stories sort of gave me hope that I could get through it, I guess and that it wasn't going to be all doom and gloom*’. (RN371) ‘*So, I think going and doing the champion study day with like‐minded people, and just went, “Oh god there's more of me out there. It's okay*”’. (RN838) For some, the programme was a helpful short‐term solution, as either a safe entry point to the system and an initial person to talk to until other psychological services became available, or to complement other identified supports, ‘*I was seeing a therapist on the outside. But having somebody that you feel maybe some sort of rapport with, like they understand your role was was a big thing for me approaching the programme. Yeah, yeah. It's different when you talk to us psychologists or psychiatrists, who's not a nurse. There's the trust, but it's different*’. (RN891) Participants felt it helped to talk to someone, the structured (or less‐structured) short sessions, pace and pathway were helpful, it felt tailored, and it tapered off nicely while retaining a safety net and sense of being able to go back if needed, ‘*So and then I think as the intensity started dying down, things started becoming less crisis, more change, more change, and so flux, the period of like a period of flux, where it's like, things are changing, you're finding a new normal. You know, I think they started cutting back on a bit more on the counselling. They always left the open… I think we ended with the offer being open, if you ever need it, you can always call us. […] I will always be grateful for the service. Like, it was an amazing thing. And it kept me sane, kept me being able to be like, figure out*’ (EN81) Others would have preferred their first point of contact to have been someone more nurturing, or a more tailored programme, ‘*I was never given sort of like a tailored programme…And I think that's something that maybe I should have had, like I should have, you know, maybe you should have had a tailored programme, and they should have been, like, you know, maybe you should come back and see us in two weeks or three weeks, or, you know, sort of setting like keeping that relationship going between client practitioner sort of situation, I think, would have been helpful for my case, rather than me having to, you know, make those appointments myself, because, you know, I might, you know, not I mean, I'm, I was obviously very determined and passionate and really wanted to get the best case for myself. So, I was proactive in booking those appointments. But I think a lot of people might not have that strength to do that themselves. And I think, particularly if they're in situational crisis, they might not feel the energy or the, you know, the drive to be sort of booking themselves in and things like that*’ (RN371) ‘*… and then I guess the I feel like they might, you know, for people who are a bit hesitant to get involved, you know, having a, an example timeline, or a plan that describes, you know, what a usual course, might look like in terms of duration, and, you know, what you can expect, might actually help sort of, use a bit of structure to help a person understand what they're getting into*’. (RN280)
Theme 3: Ways of working	Sub‐theme 3.1: Accessibility and inclusion	There was a sense of importance for clinicians in working within a service that is free, accessible, separate from the workplace, confidential and there was a sense of this contributing to social justice, ‘*is accessible to everyone regardless of their capacity to pay, so it's free… So for me, there's a real social justice component to this programme… this service is absolutely tailor made for nurses, and has a deep understanding of not only the issues that an individual might be facing, but the sort of environmental and cultural context in which it occurs … that arm's length, and that absolute disconnect between ourselves and work helps us to create a stronger therapeutic alliance*’. (clinician) Programme participants highlighted the importance of having services available nearby and in‐person, particularly for those in regional centres, ‘*But certainly, if it was, if it was expanded, they could probably have, you know, offices in major regional towns or something. […] I wanted to meet in person, because of my past experience with counsellors. Yeah. So I, I was prepared to travel to the [city] to meet with my support person*’. (RN1170) The challenge of accessing services in the country was the reality, ‘*I work in the country; I live in the country. It is harder to get any sort of service up there. […] But it's just different in that there's more isolation, I think, in the country. And, yeah, I think there is more benefit, because we struggle*’. (EN31) Clinicians thought there were opportunities to enhance cultural awareness and competence and to increase diversity among the clinician team to support inclusion which also extended to challenges matching programme participants with clinicians based on their strengths and knowledge which was not always feasible within the small team, ‘…*the lack of diversity in the team. Yeah. So diversity of all descriptions. So you know, cultural diversity is lacking. Probably age diversity is lacking it, you know, every sort of, I guess, minority is lacking. So diverse diversity, I think is something that definitely needs to be addressed*’ (clinician) ‘*I think, like, moving with the times, like, we really do have to up the game on a few things that are changing … there are a lot of things that are now in our attention, or in our consciousness that we would need to be aware of and need to start kind of addressing. Yeah, I mean, there's, there's so much we could be doing, like, indigenous health or First Nations, nurses, and midwives, you know, that, like, there's so much stuff we could be doing*’ (clinician) From the perspective of a participant, the programme was perceived to be inclusive and non‐judgemental, ‘*I guess the first thing was just, you know, knowing that that programme was there, and that I could go and talk to somebody, you know, if my if sort of, like, if that I could talk to somebody if my if the trying to phrase it here, just if the other support networks, were not sort of working, that there was like somebody else that I was, you know, really able to go to? Yeah, I found it inclusive, I found they were very sort of, you know, open to my problems that they you know, weren't judgmental, they made me feel like, you know, what I was saying…They made me feel like my, you know, like my concerns my issues mattered. Like they that they weren't kind of dismissed as something as something trivial and just you know, go back and back up and you'll be okay. So I was taken seriously Yes, I was taken seriously. So yeah, I guess I guess all those all those things. Yeah*’. (RN51) For some participants, the initial matching with a clinician was perceived to be good, they felt they were seen quickly, and they appreciated the consistency in continuing to work with the same clinician, ‘*one person was very, yeah, was very valuable. Having continuity. One person I know, it's not always practical. But certainly for me that continuity, because I didn't have to retell my story in a sense*’ (RN1170) Others would have preferred their first point of contact to have been the nurse or midwife, ‘*when I first rang up, and kind of, I just, I didn't realize that I was gonna be speaking to a receptionist first off, and so I've sort of started to tell my story and why I wanted some support. And she said, “I'll take your information and get someone to call you within 24 hours.” So, you know, there's that moment where you've plucked up the courage to actually talk to somebody and then have to wait again, which I understand now is part of the process. And when I recommend other people to do go or engage, I'll say what to expect that you'll when you ring, you'll get a receptionist. They'll take brief information, and then a counsellor will ring you back within 24 hours*’. (RN/RM224) Participants engaged with clinicians via the phone, online or in‐person, and this was described as working well for most, ‘*That was valuable too, that I had access to the programme* via *the phone. …anytime I needed help, and I was given in a very timely manner, a support time, so I didn't have to wait a long time to get help. And that was really valuable, too. Because often when you need help, it's often immediate that you need it … the support person was really valuable to me. And … accessible…I was just so amazed at the programme*’. (RN1170) Engaging was perceived easy for most, who liked the accessibility across these mediums, although meeting in person was a preferrable option for some, ‘*I was given the opportunity to meet, that was great. I do appreciate the one on one. I'm at the pointy end of my career. I'm one of those people I look, I think it's why I'm a nurse, I like human contact. I like interacting with people. These Zoom meetings are great, and they're convenient. But it just doesn't provide what you can have when you're in the same room with another person*’. (EN31) Clinicians shared the value of recent extensions of communication mediums to support online, phone and in person consultations with programme participants who might not otherwise have access, increasing flexibility and availability to meet, particularly where appropriate in‐person meeting space is limited, ‘…*think the fact that now, because of COVID, actually, we've got a lot of people are just doing it on Teams. So you know, you can, it's just much easier. So we've got a much broader reach as well*’ (clinician) ‘*…people would probably much prefer to just do a session on Microsoft Teams than drive all the way in to an office, you know, so I haven't had anyone in the whole almost year have been here request to see me in person*’. (clinician) ‘…*this phone based and, and yeah, electronic e‐based tele has changed things dramatically. And I think overall it has increased. If it has any advantage, it has the advantage of access*’. (clinician) Participants found the open‐ended availability of sessions valuable, ‘*that was an amazing benefit. That it's free access. Yeah. And it seemed like I wasn't limited to say six. You know, like some counselling, you're limited to certain sessions. At no time was I told you're not allowed to have any more. So that was really valuable as well*’. (EN81) The programme being cost‐free was also important for many, ‘*It's also valuable, that it's not costly. Yep. So that is a really was fantastic. You know, you know, you just have to be part of the union to access it. So that was an amazing benefit. That it's free access*’. (RN1170) Extending the service was considered important for reach with the support of strong resourcing to sustain workforce while maintaining team cohesion, keeping sight of programme intent, and maintaining respect and confidence built by the organization over the years, ‘*I don't know whether it there might be capacity for it to become more than an entry service. I know, I know, the team does run the other, you know, they do the promotion, I'm aware they run groups for drug and alcohol rehab, and this sort of thing. But I just wonder if there would be greater capacity to employ people… who, you know, are able to provide more than an entry level type of service, actually, you know, for those sorts of people who are waiting six months for psychology, you know, maybe something, someone … to sit on the team and actually be able to work with people and their trauma on a deeper level, so I wonder if that is another maybe another element to add to what we're able to offer*’. (clinician) ‘*so I think, yeah, maybe expanding more … offering like, say, an online support group for specific cohorts like grads or students, like they've got at the moment, they're running a support group for people with addictions once a week. […] I feel like always that lens of trying to grow, improve, make the service better, you know, encompass all aspects of health*’. (clinician)
	Sub‐theme 3.2: Complexity and crises	The goal setting, knowledge, and advice were felt to be practical and helpful, ‘…*it was very practical [advice], because that's what I needed. I didn't need you know, meditation and you know, all that. I needed practical advice. Yeah. And [the programme clinician] was able to steer me in the right way… that's why I was very happy to talk about the programme. Because it actually was the most valuable thing through my whole process. That was the most useful that I'd found*’. (RN1170) Developing a sense of knowledge and the skills to continue on without the programme was progressive, ‘*…and then towards the end, I had enough knowledge to then start accessing my own self education in what I was dealing with, so she was the catalyst for then me going on to help myself as well, which I couldn't have done without her*’. (RN1170) ‘*And they get you through that horrid space, and then work with you. And then go, you know, you've actually got this and they give you the reins back. And they never take the reins away from you*’. (RN838) For one RN, the active engagement in addressing problems with the support of the clinician was challenging, ‘*I thought overall, it was quite a quite a nurturing setup. But I also thought they asked me some questions which were quite, you know, thoughtful, and required me to, you know, agonize over the answers like this, is, it's not all Tea and Sympathy. And I think that's good. If you struggling to nut out a problem, you can go and talk to your neighbour, if you just want Tea and Sympathy. But if you really want to move on, then you need to be seeing somebody who has a few skills and can stretch you a bit. Otherwise, you might as well talk to your friends*’. (RN453) Clinicians described the importance in the flexibility and autonomy in building therapeutic relationships with participants, drawing from their strengths across a range of knowledge and experiences such as counselling, adapting to the person's unique situation whether this be a single meeting through to intermittent meetings across years as different participant needs arise, ‘*I mean, we can we hold people for quite a long time. And that's great that we're given autonomy to do that. And, and some people for whatever reason, just can't reach out for that next step of seeing a psychologist…*’ (clinician) ‘*I guess the rapport you build with the consumer of the programme, whether or not they felt listened to heard validated, you know, understood, all of that kind of stuff. And often, you know, it was the listening that they were after they wanted somebody to hear their story and to empathize with their story and to understand what was going on for them. And, you know, often that can't be easily understood and done in just one session, you know, in one sort of 50‐minute session. So, you know, often people felt that they had more to say, or more to follow‐up on…*’ (clinician) Yet also, understanding and setting boundaries as to what the service does offer, and what it does not, was a common theme, suggesting different ways of working with participants who are also engaging with other health professionals such as psychologists or who may need referrals to alternative support services, ‘*…say, really clearly, you do understand I'm not a psychologist… we can refer you*’ (clinician) The programme and case management model did not suit the needs for all, with some nurse and midwife participants suggesting their needs were too complex, ‘*I was seeing so many other professionals at the same time, they couldn't find anything. It was unrealistic, you know, for the [Programme] to find the answers*’ (EN525) Others wanted faster contact, ‘*…I got the impression that the person who picked up the phone and was making those arrangements is actually putting a bit of thought into it and that was really, that was really nice that I got to stick with someone, but also someone was quite earnest in what they were doing and I appreciated that. And I think when, when [they] did say, … it's going to be a couple of weeks until you can have a chat to them was a bit like, oh, a couple of weeks isn't great, because I sort of felt pretty ordinary at that stage. So I guess, you know, sort of something more timely would have been ideal, but I think that if it was, if, if the situation was dire, then there's obviously other resources out there that would meet that need. Yeah*’. (RN280) For some programme participants, more time with the clinicians or stronger linking with (or referrals to) other services was suggested to be a helpful option, ‘*So I guess maybe, if there were psychologists, or someone linked in, because I only saw nurses. If they had a very easy referral process into someone else, and especially what especially because it was, it's work related. And that's kind of why I went to the programme, because I was like, I need to understand why I behave the way I do when I walk into my workplace. It never used to be like this. Yeah. And so I think maybe someone who had a little bit more of experience. So psychologists or a psychiatrist, a bit more experience with the trauma would have been really helpful*’. (RNRM492) Misconceptions of the programme as a crisis service were also expressed, ‘*… it's a crisis. Yeah. And it was there to sort of get me into a secure state. It was basically to get me tided over into like, you get other services involved. And they made that very clear when you started out. You know, it's a crisis service*’. (EN81) Boundaries of the programme were felt to be clear, particularly in terms of integration and engagement with other services, ‘*I had been having issues with depression [and] anxiety over the years, and had always been using a psychologist. And that was very helpful. But I also thought I might benefit from having a person with a professional knowledge of, you know, the issues that were occurring because some of the issues were actually related to people that I was working with, so I thought it might be beneficial. [the programme clinician] was very careful. [They] knew that I was seeing a psychologist so that things that we covered, were not things that would be covered by the psychologist. [They] didn't want to be impacting on those boundaries, wanted to be very professional about that… [They were] very careful in that respect. [They] didn't want to be overstepping it, and sort of maybe impacting the information or the, the knowledge, or the whatever I was working with the psychologists. Yeah. Yeah*’. (EN594) Recognition of boundaries was echoed by clinicians, who were cognizant of the scope of the programme, and report adapting their support to fit the needs of participants and their individual needs, within the constraints imposed by the mental health care system more broadly, and to function as a safe entry point into the mental health care system, ‘*During those first few sessions, [I] try to link [the participants] into, you know, other more long term. So it's so … I view us as an entry service. So we can do, you know, obviously, we can work with them for a short term. But if they sort of need sort of a psychological input, for example, so they might need to be referred to a psychologist because their needs are greater than this service is set up to, to offer, we would start doing that in the first few sessions. But having said that, a lot of nurses recently are saying that they can't get in to see psychologists because of the wait…. And there's something ridiculous like a six month wait for a psychologist, so I'm really just […] supporting and not, you know, doing any sort of deep work with [the programme participant] because that's not the job of this…the service*’. (clinician) The focus on mental health support was valuable for many, but one RN was also hoping for career guidance, ways of getting back to the workplace or profession, and would have liked a more tailored programme, ‘*I still don't have the answers about how I could return work. That was clear, actually, that that was one of the reasons why I went up to ask how can I return to work? At that point in time, I was thinking, well, how can I return to [acute care] nursing […] And I was hoping that there was some sort of programme that could guide me back to work. Yeah, but it was not. There was nothing we definitely didn't talk about that*’. (RN891) For some, shifting the conversation from individual level, to situating problems within the broader systemic context of the health workforce and culture, was felt to be an important and unrealized step into the more political or advocacy context, ‘*I think it's a great initiative. And I think it was helpful to me. But I also felt though, and continue to feel that all of the initiatives that are put in place, still put a lot of personal responsibility onto people for things that are systemic problems. And I don't want to sound overly, overly dramatic or like, Like I'm putting together things that that perhaps don't belong together in terms of, you know, absolute seriousness, but I feel like in all of these things, the [programme] and the all the wellness stuff rolled out at work, there is still an absolute inability to look at the structural issues. And therefore I feel like there is some level of victim blaming that I'm meant to be managing a system that now is actually thoroughly broken. It was quite broken before. And now it's thoroughly broken. And we are going to continue to patch it up with stuff. That is, I think, in some ways, well meaning, but sometimes I feel like yelling at people, can you not put us in this situation in the first place, there must be people for whom this is actually their job to fix this. And by that I mean state and federal governments. And I mean, the population at large needs to work out what choices they are making, when they don't want to pay tax. You know, they really want to a lot of stuff that they have not wanting to pay for. And I think it's a hard thing to do. But it's a conversation that I think nurses take a lot to become politicized. And I see them listening to all of this and saying, oh, you know, we can go and get a wellness box, or they're, you know, putting on donuts today or there's massages, I want to scream. I really, honestly want to scream*’. (RN453) Others felt that while the programme did not provide all the answers they still felt comfortable being there and think it is a great programme, ‘*even though I didn't get out of it, what I needed was, I think it was a really great programme. And I remember reading about it and just thinking…how have I not known about it? How [is] this not in every state? Like I remember being really impressed with, you know talking about the mental health*’ (RNRM492)
Theme 4: A great day at work	Sub‐theme 4.1: Leadership and organizational culture	Clinicians choose to work in the role for a range of personal reasons, from feeling passionate for nurses' and midwives' health and wanting to be more proactive in supporting the workforce, through to enjoying the work and diversity in the role and keeping a hand in nursing. Leadership and management were felt to model inclusion, support, valuing, responsiveness, and transparent communication, ‘*I think we've got good leadership, I think we're responsive to the needs of what's happening to Victorian nurses*’. (clinician) ‘*They're good leaders, but it doesn't feel that they're managing like they're not controlling or micromanaging or making like decisions without [consultation] … it's always in consultation with the staff, like ‘what do you guys think? let's make collaborative decisions’…quite extraordinary leaders, there's really incredible leadership and the leadership is what makes a difference in organizations* … ***just love that leadership**, really just put that in bold letters in your report*’. (clinician) This extraordinary leadership was felt to be complemented by the team dynamic and fit. ‘*The key strength for me is the team. And I think it's easy to be flippant about it. But we've got a really positive work environment, which to do the work we do, I think is a key. And because of its positive work environment is based on the team members*’ (clinician) ‘*…we bring our values, core values of the organization into our discussion around, you know, what, have you seen, how have you experienced any of these values, so, we can acknowledge each other if we feel like we've been heard or supported or cared for … actually bringing those values to life, you know, they're not just stuck up on the wall…that's really important*’. (clinician) From an individual and team perspective, while some clinicians felt well supported and team connection was strong, others felt there was good support but an opportunity for more in‐person connection to develop a sense of belonging. ‘*it would just be good to for our team to get together a bit more regularly*’ (clinician) ‘*it would be nice to just be around people like in the same space and to be able to say, Oh, this is going on, like you learn from people when you're around them, rather than having to, like, make a phone call, or you're just more likely to have that casual kind of conversation. And also, I think, for me, just getting to know people in the team is beneficial to that sense of belonging and feeling connected. So having yeah, having more time, physically within the team, rather than me just working by myself at home. I mean, I like working from home, but I think the downside of that is that we miss out on the team bonding team building*’ (clinician) Clinicians embraced having space to explore innovation and improvements to make an authentic difference to participants and for themselves, offering suggestions such as building on existing educational opportunities to integrate further formal clinician education, structured case study reviews and external clinical supervision for all, ‘*having case presentations, which I think personally are the, if they're done properly, with people being prepared. I think it's the best sort of form of education, because listening to what people actually do*’. (Clinician) ‘*better at, I think, is the regular case review and structuring a bit better, rather than just “Has anyone got anything to chat about?” and which is good, but to have actually maybe just have a structured case review once a fortnight or something like that to allow that space to be able to go “I'd really like to put this all to the team in a formal sense”*’. (clinician)
	Sub‐theme 4.2: Autonomy and flexibility	Being able to work autonomously in an adaptable and person‐centred model of care was felt to be valuable, ‘*can get creative with it … it's very dynamic… it's very person centred, you know, one person might want specific strategies about how to help their physical health, and someone else might just want to vent and talk about what's going on…you can judge it based on each person's kind of life situation*’. (clinician) Clinicians enjoy and appreciate the collegiality, support, flexibility, and connection among the Program team, ‘*it's just the engagement with the participants, the nurses and the midwives, and when you really feel a great day would be when you really feel like you're making a difference, and they're feeding that back immediately to like, sort of things like relief or just feeling heard […] having participants like that gives me a lot of satisfaction at work [*…*] the nursing workforce needed support, and so if I can play a part in that, so that kind of inspired me […] one of the big things is flexibility, I have autonomy, but I have a team and support as well. So the flexibility to do as many sessions as I think they need or may not need. Yeah, that's huge. So in terms of the model, I think it's got good structure and a good team. But individually as a clinician, there's a lot of flexibility to work with clients*’ (clinician)

Throughout the four themes of ‘Preparing to connect’, ‘Shared experience’, ‘Ways of working’ and ‘A great day at work’, participants' and clinicians' experiences of engagement with the programme and the case management model have been elucidated. Experiences were largely positive and there were many highly regarded attributes of the programme. These attributes are important to consider as the programme continues to evolve. Examples of these highly regarded attributes included the shared experience of clinicians and participants which supported a common understanding, and effective programme leadership, autonomy and flexibility which supported clinicians in their role. There were also opportunities for strengthening the programme identified across all subthemes, such as programme promotion to reach particularly vulnerable populations, considering participant expectations around complexity and crises, and extending diversity among programme team.

### 5.4 RQ4: What are the experiences and perceptions of broader programme stakeholders?

The stakeholder cross‐sectional survey (Study 4) addressed RQ4, *What are the experiences and perceptions of broader programme stakeholders?* Specifically, we sought to investigate (1) key referrers' experiences and knowledge of the Program, any new mental health and psychological well‐being supports and resources they would like to see developed, and their perceived future intent to refer, and (2) key referees' experiences and knowledge of the Program and any suggestions for further mental health and psychological well‐being supports and resources they would like to see developed. Findings are reported first, for the range of ways respondents engaged with the Program, second, for respondents who referred or recommend nurses/midwives to the Program, third, for respondents who are referred Program participants and provide services to these nurses/midwives and finally, overall perceptions of all wider stakeholders of the Program.

Thirty‐nine respondents (27% response rate) consented to participate. Of these, 6 (15%) did not complete the survey in its entirety. Three respondents indicated that they had not heard of the Program. Of those that had heard of the Program, 26 reported having referred or recommended nurses/midwives to the Program in the past; 5 reported having provided services to participants of the Program; 21 reported having shared information about the Program with colleagues, friends or staff; and four reported not having engaged in any of the three ways reported above, despite having heard of the Program.

Of the 26 stakeholders who reported having previously referred or recommended nurses/midwives to the Program, 24 went on to answer additional questions about their perceptions of, and satisfaction with, the Program. Four reported having referred friends, 12 reported having referred colleagues, 13 reported having referred staff members/employees and 13 reported having referred their patients or clients (note, it was possible for participants to select multiple responses here so sums do not add up to 26). In terms of the primary organization at which referrers work, most reported being primarily employed at industrial or professional organizations when referring nurses/midwives (*n* = 15), followed by tertiary health care organizations (*n* = 6), primary health care services (*n* = 2) and peak nursing/midwifery bodies (*n* = 1). Primarily stakeholders decided to refer to, or recommend, the Program due to it being an independent organization (*M* = 9.63, *SD* 0.65) and confidential (*M* = 9.63, *SD* 0.72) (see Supplementary File 9 for a summary of all features influencing referral and recommendation).

Respondents who had previously referred or recommended someone to the Program were invited to leave comments about their decision to do so. The most frequently represented factors influencing respondents' decisions were the programme's reputation for good outcomes (*n* = 6), for example, one stakeholder suggested ‘*I find that nurses and midwives really benefit…*’, followed by clinicians' knowledge of the profession and industry (*n* = 4), for example another stakeholder suggested ‘*it is so wonderful for them that the Counsellor has an underpinning knowledge of nursing/midwifery…*’. Experiences of stakeholders when referring to, or recommending, the Program were largely favourable. Most respondents had a good idea of what would be involved (83% either agreed or strongly agreed), found it easy to do so (100% either agreed or strongly agreed) and found it easy to find information about how to refer (87% either agreed or strongly agreed).

All survey respondents who indicated they had previously heard of the Program (*n* = 33) were asked a series of questions about their satisfaction with the Program. The possible range was 0–10, with a score of 10 being the most favourable response, and an alternative option ‘I don't know/I am not sure’. Those who selected the response option ‘I don't know/I am not sure’ were excluded from analysis for individual items. Satisfaction was highly favourable with all responses either 9 or 10. Respondents were invited to enter up to three comments about the best aspects of the programme. Twenty‐one respondents left three comments, an additional two respondents left one comment. The content area of ‘By nurses and midwives, for nurses and midwives’ was most frequently reported (*f* = 17) with one respondent suggesting ‘*Nurses and Midwives are speaking with a qualified Counsellor who understands what they do and the environment that they work in*’, followed by free (*f* = 14), with one respondent suggesting ‘*Free—my members are sometimes facing termination/DV [domestic violence] / addiction and accessing resources that are free is a huge benefit to them*’ and confidential (*f* = 14) with one respondent suggesting ‘*Confidentiality is very important; in a rural town everybody know everybody and their business to boot so it is extremely difficult for anyone to access services without someone seeing you going in or coming out, you then become part of the gossip*’. In summary, stakeholder experiences and perceptions of the programme were highly favourable.

### 5.5 Joint display of integrated findings

Across four studies, four research questions have been addressed. A joint display of the findings is presented in Table 5.

**TABLE 5:** Joint display of main study findings informing research questions.

Research question	Contributing studies	Main findings
RQ1: What are the characteristics of nurses and midwives who engaged in the programme?	Studies 1 and 2	20% (*n*, 23) reported stress in the severe‐to‐extremely severe category; 22% (*n*, 25) reported psychological distress in the moderate‐to‐severe category
RQ2: What is the effectiveness of the case management model on the well‐being of nurses and midwives?	Study 2	The effect of the programme on participant well‐being over time was not significant
RQ3: What are the experiences and perceptions of nurses and midwives engaging in the programme as participants and clinicians?	Studies 1, 2 and 3	The community‐based psychological health and well‐being programme for nurses and midwives was found to be an important and highly valued resource for nurses and midwives. Experiences of nurses and midwives engaging with the programme were highly positive and strong attributes of the programme included (1) shared professional experience of clinicians and participants which supported a common language and facilitated understanding, and (2) effective programme leadership, and autonomy and flexibility in the clinicians' role which enabled and supported a positive working experience.
RQ4: What are the experiences and perceptions of broader programme stakeholders?	Study 4	All stakeholders reported high satisfaction. Participants considered the programme being ‘by nurses and midwives, for nurses and midwives’ critical to the programme's success and value.

Abbreviation: RQ, research question.

## 6 DISCUSSION

Our objectives were to evaluate a community‐based psychological health and well‐being programme for nurses and midwives to determine the (1) psychological health and well‐being characteristics of nurses and midwives engaging in the programme, (2) effectiveness of the case management model on the well‐being of nurses and midwives, (3) experiences and perceptions of nurses and midwives engaging in the programme as participants or service providers and (4) experiences and perceptions of broader stakeholders of the programme. Across the four studies of this evaluation, we have identified experiences and perceptions of nurses and midwives who engaged in the programme as participants, clinicians and wider stakeholders.

Burnout is a major concern in the health workforce, both pre‐, during and post‐pandemic (Galanis et al., 2021; Kurtzman et al., 2022). Risk factors for burnout include increased workload and inadequate resources. Notably, burnout levels were higher in the present study compared to a study of Australian midwives in 2017 (Creedy et al., 2017). Furthermore, levels of burnout were, statistically, significantly higher in the cohort who more recently participated in the Program compared to those who had engaged with the Program earlier (i.e. prior to 2020). It is possible, compared with both the midwives in 2017 and the pre‐2020 cohort, higher levels of burnout were reported due to the impacts of COVID‐19. COVID‐19 had widespread negative impacts on the psychological well‐being of nurses, particularly increasing levels of burnout (Armstrong et al., 2022). Similarly, work well‐being across several domains was significantly lower in the cohort who had participated in the Program more recently, compared to those who had participated prior to 2020. In the present study, work‐related burnout was associated with low levels of work well‐being across numerous domains. Focusing on enhancing work well‐being (e.g. job satisfaction, work happiness and work strengths use) may reduce health professionals' flight risk more than strategies to reduce ill‐being (such as burnout) alone, given the strength of the relationship between flight risk and work well‐being (*r* = −.76) is twice that of the relationship between flight risk and burnout (*r* = .49) (Jarden et al., 2022). These findings may indicate that those currently engaging with the Program are experiencing greater levels of work‐related burnout and targeting areas of work well‐being may provide a mechanism for addressing burnout.

Although the sample predominantly reported normal to mild levels of both stress and psychological distress, over 20% of participants reported stress in the severe‐to‐extremely severe category, and psychological distress in the moderate‐to‐severe category. COVID‐19 may have influenced these results; pandemic‐related stress is not a new finding. In a sample of 767 Australian acute care nurses, 17.7% reported pandemic‐related stress (Aggar et al., 2022). Similarly, Hitch et al. (2023) surveyed Australian allied health professionals (including podiatry, speech pathology and physiotherapy, but not nursing/midwifery) over three timepoints between May 2020 and December 2021, and found that stress, as measured with the DASS Stress scale, significantly increased across all intervals, with 15% of the sample reporting severe‐to‐extremely severe stress at the final timepoint. Pre‐COVID‐19 data from 450 mental health nurses in Australia from 2017 to 2018 found 7% were in the severe to extremely severe category (Delgado et al., 2021). In our current study, participants of the Program reported higher levels of stress than the sample of nurses' pre‐pandemic, however, not as severe as the sample of allied health professionals during the peak period of the pandemic in Australia. An important consideration for community health and well‐being programmes is the inclusion of interventions to address stress, such as mindfulness‐based stress reduction (Cohen et al., 2023; Lee & Cha, 2023). Although equally important is encouraging health care organizations to authentically engaged in preventative and health‐promoting initiatives (Adam et al., 2023).

Strengths use (but not strengths knowledge) was positively associated with some indicators of general well‐being, including flourishing (*r* = .479) and life satisfaction (*r* = .549), and with most of the work well‐being domain indicators. This finding is consistent with literature reporting positive associations between strengths use in occupational settings, and work engagement and well‐being (Bakker & van Woerkom, 2018). Resilience has been identified as a trait that assists people to manage and overcome stressful situations. For example, Rushton et al. (2015) found inverse associations between resilience and burnout, and resilience and stress and positive associations between resilience and hope. Delgado et al. (2021) reported high correlations between workplace resilience and psychological well‐being. Respondents in the present study reported lower levels of resilience than a sample of Victorian nurses during the 2020 pandemic (Jarden et al., 2022), assessed with the same measure. Health care professionals who report high levels of resilience are those with more experience and acceptable pay and workload (Castillo‐Gonzalez et al., 2024). Reported levels of resilience were likely impacted by the significant increases in workload experienced during COVID‐19 (Qureshi et al., 2022). Nurses and midwives individually and collectively have agency and can proactively strengthen resilience, however, building resilience requires a multifaceted systems approach. Community health and well‐being programmes are an important part of this system. Focusing on resilience as a whole of career strategy (Henshall et al., 2020) reinforces the importance of health and well‐being community programmes working with tertiary education providers delivering entry to practice programmes.

A surprising aspect of this evaluation was related to the quantitative findings of the longitudinal survey, which found no statistically significant effects of the programme on participant well‐being. This was in contrast to the qualitative data (free response items from surveys and interviews) in which participants reported feeling validated and supported. Participants reported the programme was highly valued, experiences were largely positive, and there were many well regarded attributes of the programme. Several factors may have contributed to this inconsistency between the quantitative and qualitative data, discussed further in the limitations.

### 6.1 Recommendations for further research

Consistent with previous literature, the present study identified inverse relationships between resilience and stress, and between resilience and psychological distress, and positive relationships between greater resilience and greater happiness and between greater resilience and greater life satisfaction. Actively working with nurses and midwives to strengthen resilience is an important aspect for community health and well‐being programmes to retain. Extending work with educational and health care organizations to support health promotion and prevention of ill‐being is an important endeavour. Investigating potential new models for health promotion and ill‐being prevention for nurses and midwives, and measuring the effectiveness of these endeavours, is an essential next step in research.

### 6.2 Implications for policy and practice

Locally, in 2019, the Governor of the State of Victoria, Australia, formally established the Royal Commission into Victoria's Mental Health System (State of Victoria, 2021). The subsequent 2021 report highlighted the existing system was not ‘designed or equipped to support the diverse needs of people living with mental illness or psychological distress … let alone to cope with unforeseen pressures that may arise’ (p. 4, State of Victoria, 2021) and set out 65 recommendations for transformation of the Victorian mental health system. The Program is well positioned in contributing to a contemporary and adaptable mental health and well‐being system, particularly considering the commission's recommendations focusing on availability and access to treatment, care and support. This positioning of Program is opportune to not only address and prevent nurses' and midwives' ill‐being and work ill‐being, but also to promote well‐being and work well‐being. Given that around 10% of COVID‐19 cases globally were among health care workers (International Council of Nurses, 2020), the impact of COVID‐19 is embedded in this service evaluation and will inform ongoing investigation and recommendations for future preparedness.

### 6.3 Limitations of the work

The small sample size and dropout rate in studies one and two limits generalizability. This problem was exacerbated by the heterogeneous sample—some (~50% of) participants at timepoint 3 had engaged with the programme since completing the baseline survey, whereas others had not accessed services since baseline, 12 months ago. The low response rate and subsequent dropout during the longitudinal survey may indicate survey fatigue, burnout, workloads and/or speak to a bigger issue regarding whether potential participants believe participation in an evaluation will afford real change. Low survey response rates are a growing global concern contributing to a non‐response bias (Timmins et al., 2023). There was also potential for a response bias in the longitudinal survey. It was possible that participants who had a better experience with the Program were more likely to complete the survey at follow‐up timepoints. Furthermore, those participants more actively engaged with the programme may have been more likely to respond to follow‐up surveys.

## 7 CONCLUSIONS

In this programme evaluation, participants reported high regard for the Program, from the nurses and midwives who had participated in the Program, to the clinicians working within the Program, to referrers to the Program, to those who receive referrals from the Program, to the broader industry stakeholders of the Program. The importance of the Program being ‘by nurses and midwives, for nurses and midwives’—that is, clinicians' lived experience working as nurses and/or midwives—was considered not only unique, but also critical to the success and value of the Program. Given this highly valued feature, the Program was perceived to hold an important and unique place within the broader mental health and well‐being system. Both clinicians and participants identified the role, integration and boundaries of the service, either as a safe entry point to the system or as an adjunct to services provided by other organizations. Exploring ways to retain the core strengths of the Program while enhancing reach and diversity will be important next steps as the Program continues to develop.

## AUTHOR CONTRIBUTIONS

All authors have agreed on the final version and meet at least one of the following criteria: (1) substantial contributions to conception and design, acquisition of data or analysis and interpretation of data; (2) drafting the article or revising it critically for important intellectual content. **Rebecca Jarden**: Conceptualization, formal analysis, investigation, writing—original draft, writing—review and editing, visualization, supervision and project administration. **Aaron Jarden**: Formal analysis, investigation, writing—original draft and writing—review and editing. **Helena Bujalka**: Formal analysis, investigation and writing—review and editing. **Tracey Weiland**: Conceptualization and writing—review and editing. **Naomi Brockenshire**: Formal analysis, investigation and writing—review and editing. **Glenn Taylor**: Conceptualization and writing—review and editing. **Marie Gerdtz**: Conceptualization and writing—review and editing.

## FUNDING INFORMATION

This independent programme evaluation was funded by Nursing and Midwifery Health Program Victoria. The programme had no involvement in study design, collection, analysis and interpretation of data.

## CONFLICT OF INTEREST STATEMENT

Co‐author Glenn Taylor is chief executive officer of the evaluated health programme. Mr Taylor had no involvement in study design, collection, analysis and interpretation of data.

## ETHICAL CONSIDERATIONS

This mixed methods evaluation was approved by the Human Ethics Committee of the University of Melbourne (2022‐22821‐25367‐3).

## Supporting information


Supplementary Files 1.

## Data Availability

The authors confirm that the data supporting the findings of this study are available within the article and supplementary materials.
