# Supplementary material for: Community‐based health programme for nurses and midwives: A mixed methods evaluation
Source: J Adv Nurs. 2024 Jul 30;81(1):475–98. doi: 10.1111/jan.16336 (PMC11638500; doi:10.1111/jan.16336)
Supplement: Supplementary file 1 — Supplementary Files 1. [file JAN-81-475-s001.docx]

# SUPPLEMENTARY FILES

## Supplementary File 1. Survey items, measures and interview guide for the Program evaluation.

(Note: excluding previously reported validated measures such as DASS, Kessler 10, CBI, Flourishing Index)

#### **Satisfaction**

(1 = Not beneficial at all – 10 = Extremely beneficial; 11 = I don’t know / I’m not sure)

1. How beneficial has the Program been for you?
2. How would you rate the quality of the service you received from the Program?
3. To what extent has the Program meet your needs?
4. If a nurse/midwife friend/colleague needed similar help, how likely would you be to recommend the Program?
5. To what extent are you satisfied with the amount of support you received from the Program?
6. To what extent did participating in the Program help you deal more effectively with your problems?
7. Thinking about your overall experience as a participant in the Program, how satisfied are you with the services you received?
8. If you needed to seek support for similar issues in the future, how likely would you to be use the services of the Program?

#### Three best things

1. What are/were the three best things about your participation in the Program?

#### Other supports

1. Did your engagement with the Program lead you to seek different types of support or other services?
2. Please list or describe any support services that you accessed because of your involvement with the Program.

#### Goals

(0 = Not at all – 10 = Completely)

1. To what extent do you feel that the goals you have set are achievable within the timeframes you have set?
2. To what extent do you feel like you are progressing with the goals you have set?

#### Happiness

(10 = Extremely happy – 0 = Extremely unhappy; see The Happiness Measures (Fordyce, 1988) also known as the Fordyce Emotion Questionnaire. Provides a qualitative, rather than quantitative, feel of happiness.)

1. The following question asks you how you generally feel. In general, how happy or unhappy do you usually feel?

#### Satisfied with life

(10 = Completely satisfied – 0 = Not at all satisfied; see (Diener, Inglehart, & Tay, 2012). See also, (Diener, & Gonzalez, 2011). Recommended question by (OECD, 2013).)

1. Overall, how satisfied are you with life as a whole these days?

#### Satisfied with health

(10 = Completely satisfied – 0 = Not satisfied at all; Informed by the Sovereign Wellbeing Index, see (Human Potential Centre, 2013).

1. In general, how satisfied are you with your health?

#### Satisfied with diet

(10 = Completely satisfied – 0 = Not satisfied at all; Informed by the Sovereign Wellbeing Index, see (Human Potential Centre, 2013).

1. In general, how satisfied are you with your diet?

#### Satisfied with sleep

(10 = Completely satisfied – 0 = Not satisfied at all)

1. In general, how satisfied are you with the quality of your sleep?

#### Satisfied with physical activity & exercise

(10 = Completely satisfied – 0 = Not satisfied at all)

1. In general, how satisfied are you with your level of physical activity and exercise?

#### Job satisfaction

(10 = Completely satisfied – 0 = Not satisfied at all; refer to European Social Survey (European Social Survey, 2006) and British Household Panel Survey (1991).)

1. All things considered, how satisfied are you with your present job?

#### Work life balance

(10 = Completely satisfied – 0 = Not satisfied at all; refer to use by Office for National Statistics in UK.)

1. How satisfied are you with the balance between the time you spend on your paid work, and the time you spend on other aspects of your life?

#### Work happiness

(10 = Completely happy – 0 = Not at all happy)

1. How happy do you generally feel at work?

#### Work relationships

(10 = Completely satisfied – 0 = Not at all satisfied; Informed by Van den Broeck, Vansteenkiste De Witte, Soenens, & Lens, (2010) and Fernet, Austin, Trépanier, & Dussault, (2013)).

1. How satisfied are you with your workplace relationships?

#### Work alive and vital

(10 = Completely alive and vital – 0 = Not at all alive and vital; Adapted from The Vitality Scale (Ryan & Frederick, 1997))

1. In general, how alive and vital do you feel in your job?

#### Work motivation

(10 = Completely motivated – 0 = Not at all motivated; work motivation is strongly linked to performance - see (Latham, 2007) or (Kanfer, Chen, & Pritchard, 2008).

1. How motivated are you in what you are doing for your job?

#### Valued by manager

(10 = Completely valued – 0 = Not at all valued; Based on (Cook & Wall, 1980)

1. How valued do you feel by your manager?

#### Job stressful

(10 = Completely stressful – 0 = Not at all stressful; Based on The Stress in General Scale, see Stanton, Balzer, Smith, Parra, & Ironson (2001))

1. On average, how stressful is your job?

#### Valued by organisation

(10 = Completely valued – 0 = Not at all valued; evaluator question)

1. How valued do you feel by your organisation?

#### Make a difference doing job

(10 = completely - 0 = not at all; evaluator question)

1. Do you feel like you make a difference doing your job?

#### Difficultly disconnecting from work

(10 = completely – 0 = not at all; evaluator question)

1. How difficult is it for you to disconnect from work when you are not at work?

#### Intend to leave job

(10 = Completely likely – 0 = Not at all likely; Informed by Weisberg (1994))

1. How likely is it that you will leave your job in the next six months?

#### Proud of work & Job inspires me

(6 = Always – 0 = Never; from the Utrecht Work Engagement Scale (Schaufeli, Baker, & Salanova, 2006). Schaufeli, W. B., Baker, A. A., & Salanova, M. (2006). The measurement of work engagement with a short questionnaire. A cross-national study. Educational and Psychological Measurement, 66(4), 701-716.)

1. I am proud of the work I do.
2. My job inspires me.

## Study 1 & 2 measures.

| Measure or Items | Description | Scoring details | Possible Range |
| --- | --- | --- | --- |
| Happiness and Life Satisfaction *(from Work on Wellbeing (WoW) assessment battery, a collection of previously validated scales, measures, & individual items)* | A question about life satisfaction and a question about happiness. | Items scored on a 10-point scale from 0 (Not at all satisfied) - 10 (Completely satisfied) | Each item is on a scale of 0-10. |
| Health and Lifestyle Factors (WoW) | Four items assessing satisfaction with overall health, diet, sleep quality and level of physical activity^‡^. | Items scored on a 10-point scale from 0 (Not at all satisfied) - 10 (Completely satisfied). The health and lifestyle score was calculated by an average of four questions (perceived health, nutrition, physical activity, sleep) and then converted to a percentage. Greater scores indicate greater health and lifestyle wellbeing. | 0 – 100. |
| Work wellbeing (WoW) | 11 of the 17 work wellbeing items from the Work on Wellbeing assessment were selected^‡^. | Items scored on a 10-point scale from 0 (Not at all satisfied) - 10 (Completely satisfied). | Items treated individually, each on a scale from 0-10. |
| Utrecht Work Engagement Scale (UWES, Schaufeli et al., 2006) | Two items from the UWES were selected^‡^. | Each item is scored on a scale of 0 (Never) to 6 (Always). | These items were treated as individual variables, each on a scale of 0 to 6. |
| Brief Resilience Scale (BRS, Smith et al., 2008) | The 6-item scale assesses the ability to bounce back or recover from stress. | Each item is scored on a scale of 0 (Not at all like me) - 10 (Completely like me). Three negatively worded items are reverse scored. | The overall score is calculated by summing the responses to the 6 questions, giving a possible range of 0-60, where higher scores indicate greater resilience. |
| Kessler-10 (K10, Kessler et al., 2003) | A global measure of distress based on questions about anxiety and depressive symptoms that a person has experienced in the most recent 4-week period. | 10 items are scored on a 5-point scale, 1 (None of the time) -5 (All of the time). | Scores for 10 items are summed, total possible range is 10-50, greater scores indicate greater level of psychological distress. |
| Flourishing Scale (Diener et al., 2010) | The 8-item scale assesses self-perceived success in areas identified as important for psychological flourishing, including relationships, meaning and purpose, self-esteem and optimism. | Items are scored on a 7-point scale from 1 (strongly disagree) to 7 (strongly agree). | The eight items are summed, and scores range from 8 to 56. A high score on the scale indicates respondents have a positive self-image in important areas of functioning. |
| Work-related burnout scale of the Copenhagen Burnout Inventory (Kristensen et al., 2005) | A 7-item scale assessing work-related burnout, or “the degree of physical and psychological fatigue and exhaustion that is perceived by the person as related to his/her work” (Kristensen et al., 2005). | Each item is scored on a five-point scale; four items are scored on a scale of Always (100) to Never/Almost Never (0); the other three items are scored on a scale of 100 (To a very High Degree) to 0 (To a Very Low Degree). One item is reverse scored. | The overall burnout score is an average of the scores for each item. A lower score indicates lower burnout. |
| DASS Stress (Lovibond & Lovibond, 1996) | A 7-item stress scale which is sensitive to levels of chronic non-specific arousal and assesses difficulty relaxing, nervous arousal and being easily upset/agitated, irritable/over-reactive and impatient. | Each item is scored on a 4-point scale ranging from 0 (did not apply to me at all) to 3 (applied to me very much or most of the time). | Sum the 7 items then multiply by 2, to produce a score ranging from 0-42. Higher scores indicate greater perceived stress. Scores are categorised as Normal (0-10), Mild (11-18), Moderate (19-26), Severe (27-34) and Extremely Severe (35-42). |
| Strengths Use and Strengths Knowledge (Govindji & Linley, 2007) | Adapted (shortened) in accordance with the Work on Wellbeing assessment battery, which uses three questions for Strengths Use and three questions for Strengths Knowledge. | Responses are on an 11-point scale from 0 (Strongly Disagree) to 10 (Strongly Agree); the scores for the three strengths use questions and the three strengths use questions are summed separately. | A total possible range of 0-30 for the Strengths Use subscale and also for the Strengths Knowledge scale. |

Notes: BRS = Brief Resilience Scale, DASS = Depression, Anxiety and Stress Scale, K10 = Kessler 10-item scale, UWES = Utrecht Work Engagement Scale, WoW = Work on Wellbeing assessment battery; ^‡^see Supplementary File 2 for further details on questions and items selected to reduce participant burden.

## Interview guide.

**Interview guide for program participants**

Introductions…

1. Are you a nurse or midwife?
2. What inspired you to become a nurse/midwife?
3. Are you still practising as a nurse?
4. What does a great day at work look like to you?
5. Tell me about how you first heard of the program.
6. Describe your first phone call/experience.
7. Describe your first experience with the clinician.
8. Describe next steps after the first conversation with the clinician?
9. When you think back to your experience with the program, tell me if/how it met your initial expectations? Why?
10. Do you have any other comments you would like to share about your experience with the Program?

**Interview guide for clinicians**

Introductions…

1. What inspired you to join Program?

What does a great day at work look like to you working as a clinician at Program?

What do you see are the greatest strengths of the program?

What do you think are the opportunities for strengthening the program?

Tell me about program: awareness/referral pathways/access/engagement/model of case management/outcomes/future.

## Supplementary File 2. Recruitment across studies.

|  | Study 1  Cross-sectional survey  (*n* = 115) | Study 2  Longitudinal survey  (*n* = 31) | Study 3  Interviews with program participants (*n* = 16) & clinicians (*n* = 8) | Study 4  Stakeholder survey  (*n* = 39) |
| --- | --- | --- | --- | --- |
| Data collection period | April-May 2022. | April 2022-April 2023. | Clinicians: October 2022-January 2023.  Program participants: June 2022-March 2023. | August 2022. |
| Participants | Nurses/midwives who had participated in the Program at any time from 2006 to 2020. | Nurses/midwives who had participated in the Program from 2020 to 2022. | Current and past program participants (2006 to 2022) and clinicians of the Program. | Stakeholders included: Chief nurses at local hospitals, Deans of Nursing at local universities/colleges that offer nursing/midwifery courses, individuals at the peak professional and organisational bodies, individuals at health services, organisations that the Program reports referring participants to. |
| Recruitment method | Direct email invitation to participant with unique survey link. | Direct email invitation to participant with unique survey link. | Clinicians: Direct email to clinicians (details were provided to the researchers by the Program)  Program participant: Direct email invitation to respondents in the cross sectional /longitudinal surveys who indicated in the survey that they were willing to be contacted for an interview. | Group email, which contained an anonymous link, to individuals and organisations that were identified as stakeholders; email list compiled from addresses supplied to the researchers by the Program, and by the researchers. |
| Research Questions (RQ) addressed | | | | |
| RQ1: What are the psychological health and wellbeing characteristics of nurses and midwives who engaged in the program | Y | Y | N | N |
| RQ2: What is the effectiveness of the case management model on the wellbeing of nurses and midwives | N | Y | N | N |
| RQ3: What are the experiences and perceptions of nurses and midwives engaging in the program as participants and clinicians? | Y | Y | Y | N |
| RQ4 Experiences and perceptions of stakeholders | N | N | N | Y |

*Notes: N = No, Y = Yes; RQ = Research Question*

## Supplementary File 3. Wellbeing and illbeing descriptives.

#### Work wellbeing

| Variable (Possible Range) | Before 2020  N=84 | | | | After 2020  N=26 | | | Total sample  N=110 | | | Mann-Whitney U test significance |
| --- | --- | --- | --- | --- | --- | --- | --- | --- | --- | --- | --- |
|  | **Mean (SD)** | **Range** | | **N** | **Mean (SD)** | **Range** | **N** | **Mean (SD)** | **Range** | **N** |  |
| All things considered, how satisfied are you with your present job?  (0 – 10)^a^ | 6.38 (2.61) | 0 – 10 | | 71 | 5.10 (2.81) | 0 – 10 | 29 | 6.01 (2.71) | 0 – 10 | 100 | .032* |
| How satisfied are you with the balance between the time you spend on your paid work, and the time you spend on other aspects of your life?  (0 – 10)^a^ | 6.14 (2.50) | 0 – 10 | | 71 | 5.45 (2.95) | 0 – 10 | 29 | 5.94 (2.64) | 0 – 10 | 100 | 0.258 |
| How happy do you generally feel at work?  (0 – 10)^a^ | 6.11 (2.69) | 0 – 10 | | 70 | 5.11 (2.78) | 0 – 8 | 28 | 5.83 (2.74) | 0 – 10 | 98 | .090 |
| How satisfied are you with your workplace relationships?  (0 – 10)^a^ | 6.41 (2.72) | 0 – 10 | | 70 | 5.97 (3.01) | 0 – 10 | 29 | 6.28 (2.80) | 0 – 10 | 99 | .528 |
| In general, how alive and vital do you feel in your job?  (0 – 10)^a^ | 6.15 (2.58) | 0 – 10 | | 71 | 4.93 (2.91) | 0 – 10 | 29 | 5.80 (2.72) | 0 – 10 | 100 | .051 |
| How motivated are you in what you are doing for your job?  (0 – 10)^a^ | 6.92 (2.54) | 0 – 10 | | 71 | 5.72 (2.85) | 1 – 10 | 29 | 6.57 (2.68) | 0 – 10 | 100 | .058 |
| How valued do you feel by your manager (0 – 10)^a^ | 5.99 (3.20) | 0 – 10 | | 70 | 5.10 (3.48) | 0 – 10 | 29 | 5.73 (3.29) | 0 – 10 | 99 | .242 |
| How valued do you feel by your organisation  (0 – 10)^c^ | 4.83 (3.03) | 0 – 10 | | 70 | 3.83 (3.35) | 0 – 10 | 29 | 4.54 (3.14) | 0 – 10 | 99 | .136 |
| Do you feel like you make a difference doing your job?  (0 – 10)^c^ | 7.37 (2.55) | | 0 – 10 | 70 | 7.17 (2.59) | 1 – 10 | 29 | 7.31 (2.55) | 0 – 10 | 99 | .725 |
| I am proud of the work I do.  (0 – 6)^b^ | 4.83 (1.16) | 0 – 6 | | 72 | 4.54 (1.14) | 2 – 6 | 28 | 4.75 (1.16) | 0 – 6 | 100 | .204 |
| My job inspires me.  (0 – 6)^b^ | 4.15 (1.47) | 0 – 6 | | 71 | 3.83 (1.42) | 1 – 6 | 29 | 4.06 (1.45) | 0 – 6 | 100 | .216 |
| How difficult is it for you to disconnect from work when you are not at work?  (0 – 10)^c^ | 4.79 (2.72) | 0 – 10 | | 71 | 5.66 (2.98) | 0 – 10 | 29 | 5.04 (2.81) | 0 – 10 | 100 | .120 |
| On average, how stressful is your job?  (0 – 10)^a^ | 6.48 (2.58) | 0 – 10 | | 71 | 7.76 (2.03) | 4 – 10 | 29 | 6.85 (2.50) | 0 – 10 | 100 | .019* |
| a. Questions from the Work on Wellbeing (WoW) assessment battery.  b. Questions from the Utrecht Work Engagement Scale (Schaufeli et al. (2006).  c. Questions suggested by Program sponsors for this evaluation. | | | | | | | | | | | |

#### Work illbeing: Burnout (CBI) summary

Levels of work-related burnout (as measured by the work subscale of the Copenhagen Burnout Inventory) in the pre-2020 and post-2020.

|  | **Pre-2020** | | | **Post-2020** | | | **Combined group** | | | **Comparator Sample** | **Mann-Whitney U test significance** |
| --- | --- | --- | --- | --- | --- | --- | --- | --- | --- | --- | --- |
| **Variable (possible range)** | **M (SD)** | **Range** | **n** | **M (SD)** | **Range** | **n** | **M (SD)** | **Range** | **n** | **M (SD)** |  |
| Burnout – work subscale  (0-100) | 47.12 (21.99) | 3.57 – 96.43 | 72 | 59.95 (21.79) | 28.57 - 100 | 28 | 50.71 (22.58) | 3.57 – 100 | 100 | 44.69 (19.23)^#^ | .024* |
| #Study of 978 Australian midwives in 2017 (Creedy et al., 2017). | | | | | | | | | | | |

#### Work illbeing: Burnout severity

Prevalence of levels of work-related burnout in the pre- and post-2020 cohorts, and for the combined sample.

|  | **Pre-2020** | | **Post-2020** | | **Combined Group** | |
| --- | --- | --- | --- | --- | --- | --- |
| Burnout prevalence | **n** | **%** | **n** | **%** | **n** | **%** |
| Low/No (<50) | 34 | 47% | 10 | 36% | 44 | 44% |
| Moderate (50-74) | 29 | 40% | 9 | 32% | 38 | 38% |
| High (75-99) | 9 | 12% | 8 | 29% | 17 | 17% |
| Severe (100) | 0 | 0% | 1 | 4% | 1 | 1% |

#### General wellbeing: Flourishing, health and lifestyle, happiness, and life satisfaction

|  | **Pre-2020** | | | **Post-2020** | | | **Combined** | | | **Comparator Sample M (SD)** | **Mann-Whitney U test significance** |
| --- | --- | --- | --- | --- | --- | --- | --- | --- | --- | --- | --- |
| **Variable (possible range)** | **M(SD)** | **Range** | **n** | **M(SD)** | **Range** | **n** | **M(SD)** | **Range** | **n** |  |  |
| Flourishing Scale  (8-56) | 44.16 (9.82) | 8-56 | 74 | 44.00 (7.47) | 17-53 | 29 | 44.12 (9.18) | 8-56 | 103 | 47.03 (5.96)^ | .387 |
| Health and Lifestyle Indicator  (0-100) | 58.70 (20.73) | 0-100 | 73 | 57.75 (14.39) | 25-92.5 | 30 | 58.42 (19.04) | 0-100 | 103 |  | .263 |
| Happiness  (0-10) | 6.40 (2.09) | 1-9 | 75 | 6.27 (2.21) | 1-8 | 30 | 6.36 (2.11) | 1-9 | 105 |  | .926 |
| Life satisfaction  (0-10) | 6.80 (2.05) | 1-10 | 74 | 6.67 (1.94) | 3-10 | 30 | 6.76 (2.01) | 1-10 | 104 |  | .399 |
| ^Jarden et al. (2022): sample of 49 nurses in Victoria during the 2020 pandemic | | | | | | | | | | | |

#### General illbeing: Stress (DASS-Stress) and psychological distress (K-10)

| Variable (possible range) | Pre-2020 | | | Post-2020 | | | Combined | | | | Comparator Sample |  |
| --- | --- | --- | --- | --- | --- | --- | --- | --- | --- | --- | --- | --- |
|  | **M (SD)** | **Range** | **n** | **M (SD)** | **Range** | **n** | **M (SD)** | **Range** | **alpha** | **n** | M (SD) | **Mann-Whitney U test significance** |
| DASS – Stress subscale (0 – 21) | 16.64 (10.20) | 0 – 42 | 72 | 20.5 (10.09) | 4 – 42 | 28 | 17.71 (10.27) | 0 – 42 | .92 | 100 | 10.48  (8.26)^ | .087 |
| Kessler-10  (10-50) | 20.01 (9.56) | 10 – 50 | 70 | 20.64 (6.67) | 12 – 33 | 28 | 20.19 (8.80) | 10 – 50 | .95 | 98 | 19.7^&^ | .247 |
| ^Delgado et al. (2021), a study of 450 RNs working in mental health setting in Australia.  &Stubbs et al. (2021), a study of 433 hospital workers (including 133 nurses) in a large tertiary teaching hospital in NSW, during the 2020 pandemic. | | | | | | | | | | | | |

#### General illbeing: Prevalence of states of stress (DASS-Stress) and psychological distress (K-10) for each cohort and the combined group.

|  | **Pre-2020** | | **Post-2020** | | **Combined** | | **Comparator Sample^** |
| --- | --- | --- | --- | --- | --- | --- | --- |
|  | **n** | **%** | **n** | **%** | **n** | **%** |  |
| **DASS Stress prevalence** |  |  |  |  |  |  |  |
| Normal (0-14) | 35 | 48.6% | 10 | 35.7% | 45 | 45.0% | 75% |
| Mild (15-18) | 9 | 12.5% | 4 | 14.3% | 13 | 13.0% | 9.3% |
| Moderate (19-25) | 11 | 15.3% | 8 | 28.6% | 19 | 19.0% | 8.0% |
| Severe (26-33) | 12 | 16.7% | 2 | 7.1% | 14 | 14.0% | 6.0% |
| Extremely severe  (34+) | 5 | 6.9% | 4 | 14.3% | 9 | 9.0% | 1.3% |
|  |  |  |  |  |  |  |  |
| **K-10 Psychological Distress prevalence** |  |  |  |  |  |  |  |
| Normal (0-19) | 43 | 61.4% | 16 | 57.1% | 59 | 60.2 |  |
| Mild distress (20-24) | 11 | 15.7% | 3 | 10.7% | 14 | 14.3 |  |
| Moderate distress (25-29) | 4 | 5.7% | 6 | 21.4% | 10 | 10.2 |  |
| Severe distress (≥30) | 12 | 17.1% | 3 | 10.7% | 15 | 15.3 |  |
| ^Delgado et al. (2021), a study of 450 RNs working in mental health setting in Australia. | | | | | | | |

#### Strengths Use, Strengths Knowledge and Brief Resilience Scale

|  | Pre-2020 | | | Post-2020 | | | Combined sample | | | Comparator Sample | Mann-Whitney U test significance |
| --- | --- | --- | --- | --- | --- | --- | --- | --- | --- | --- | --- |
| Variable (possible range) | **M (SD)** | **Range** | **n** | **M (SD)** | **Range** | **n** | **M (SD)** | **Range** | **n** | **M (SD)** |  |
| Strengths Use (0-30) | 21.22 (6.10) | 0 – 30 | 69 | 19.46 (5.86) | 6 – 30 | 28 | 20.71 (6.06) | 0 – 30 | 97 | - | .107 |
| Strengths Knowledge (0-30) | 23.62 (5.84) | 0 – 30 | 69 | 23.00 (5.44) | 9 – 30 | 28 | 23.44 (5.71) | 0 – 30 | 97 | - | .457 |
| Brief Resilience Scale (0 – 60) | 31.32 (8.46) | 1 – 48 | 74 | 31.45 (11.00) | 4 – 48 | 29 | 31.36 (9.19) | 1 – 48 | 103 | 40.81^ (11.38) | .872 |

^Jarden et al. (2022): sample of 49 nurses in Victoria during the 2020 pandemic.

## Supplementary File 4. Program participants satisfaction with program.

| Variable | Before 2020  N=84 | | | After 2020  N=26 | | | Total sample  N=110 | | | Mann-Whitney U test significance |
| --- | --- | --- | --- | --- | --- | --- | --- | --- | --- | --- |
|  | **Mean (SD)** | **Range** | **N** | **Mean (SD)** | **Range** | **N** | **Mean (SD)** | **Range** | **N** |  |
| How beneficial has the program been for you? | 7.63 (2.86) | 1 – 10 | 83 | 9.19 (1.45) | 5 - 10 | 31 | 8.05 (2.64) | 1 – 10 | 114 | .008** |
| How would you rate the quality of the service you received from the Program? | 7.96 (2.43) | 0 – 10 | 78 | 9.23 (1.17) | 5 - 10 | 31 | 8.32 (2.22) | 0 – 10 | 109 | .004** |
| To what extent has the Program meet your needs? | 7.41 (2.92) | 0 – 10 | 78 | 9.03 (1.50) | 4 – 10 | 30 | 7.86 (2.70) | 0 – 10 | 108 | .004** |
| If a nurse/ midwife friend/colleague needed similar help, how likely would you be to recommend the Program? | 8.35 (2.77) | 0 – 10 | 80 | 9.55 (1.41) | 5 - 10 | 31 | 8.68 (2.52) | 0 – 10 | 111 | .006* |
| To what extent are you satisfied with the amount of support you received from the Program? | 7.71 (2.86) | 0 – 10 | 80 | 9.19 (1.49) | 5 – 10 | 31 | 8.13 (2.63) | 0 – 10 | 111 | .004** |
| To what extent did participating in the Program help you deal more effectively with your problems? | 7.17 (2.95) | 0 – 10 | 81 | 8.81 (1.40) | 5 – 10 | 31 | 7.63 (2.71) | 0 – 10 | 112 | .005** |
| How satisfied are you with the services you received? | 7.76 (2.90) | 0 – 10 | 79 | 9.32 (1.33) | 5 – 10 | 31 | 8.20 (2.64) | 0 – 10 | 110 | .003** |
| If you needed to seek support for similar issues in the future, how likely would you to be use the services of the Program? | 7.36 (3.36) | 0 – 10 | 75 | 9.06 (2.11) | 2 – 10 | 31 | 7.86 (3.13) | 0 – 10 | 106 | .006* |

## Supplementary File 5. Correlation matrix between main study variables.


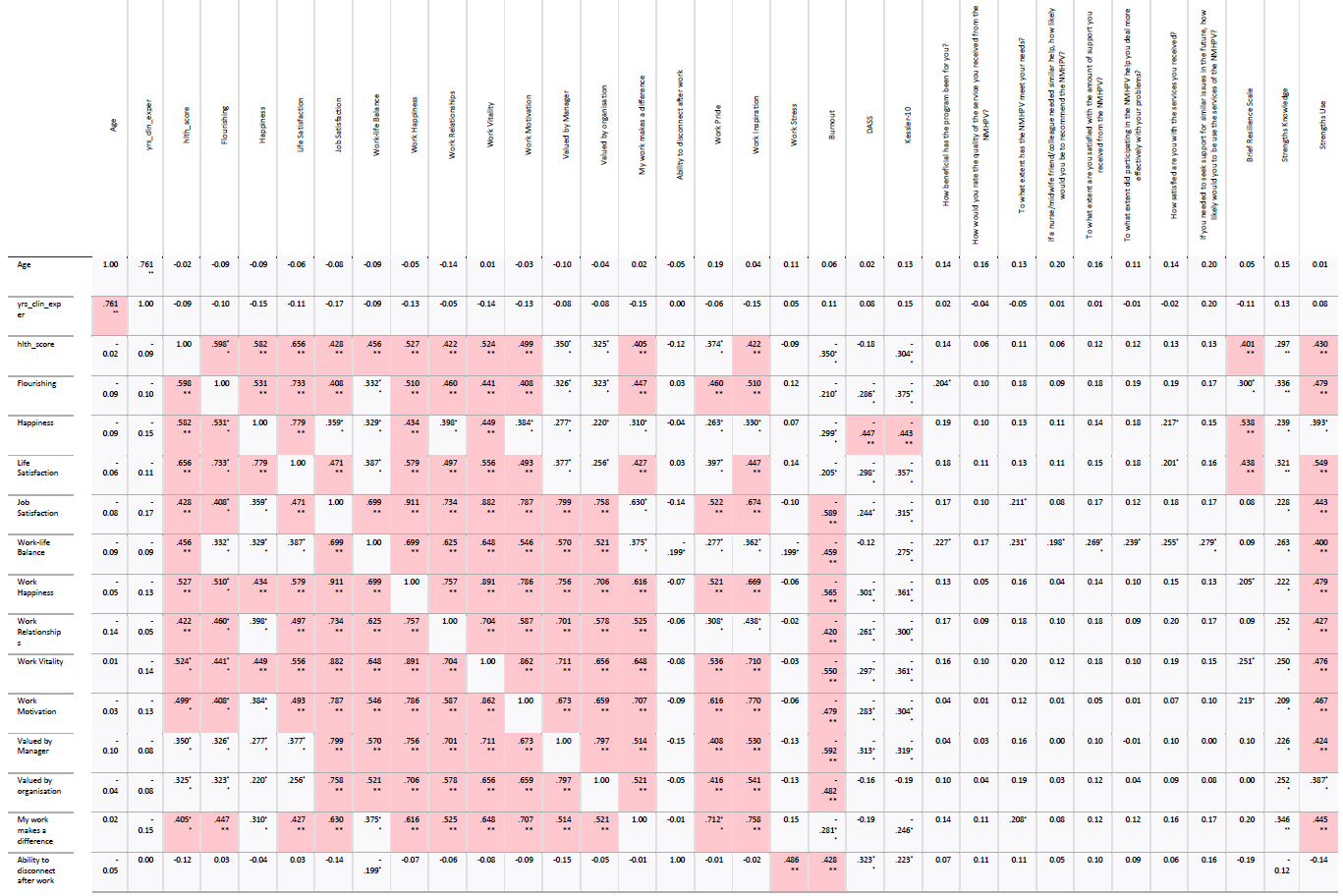


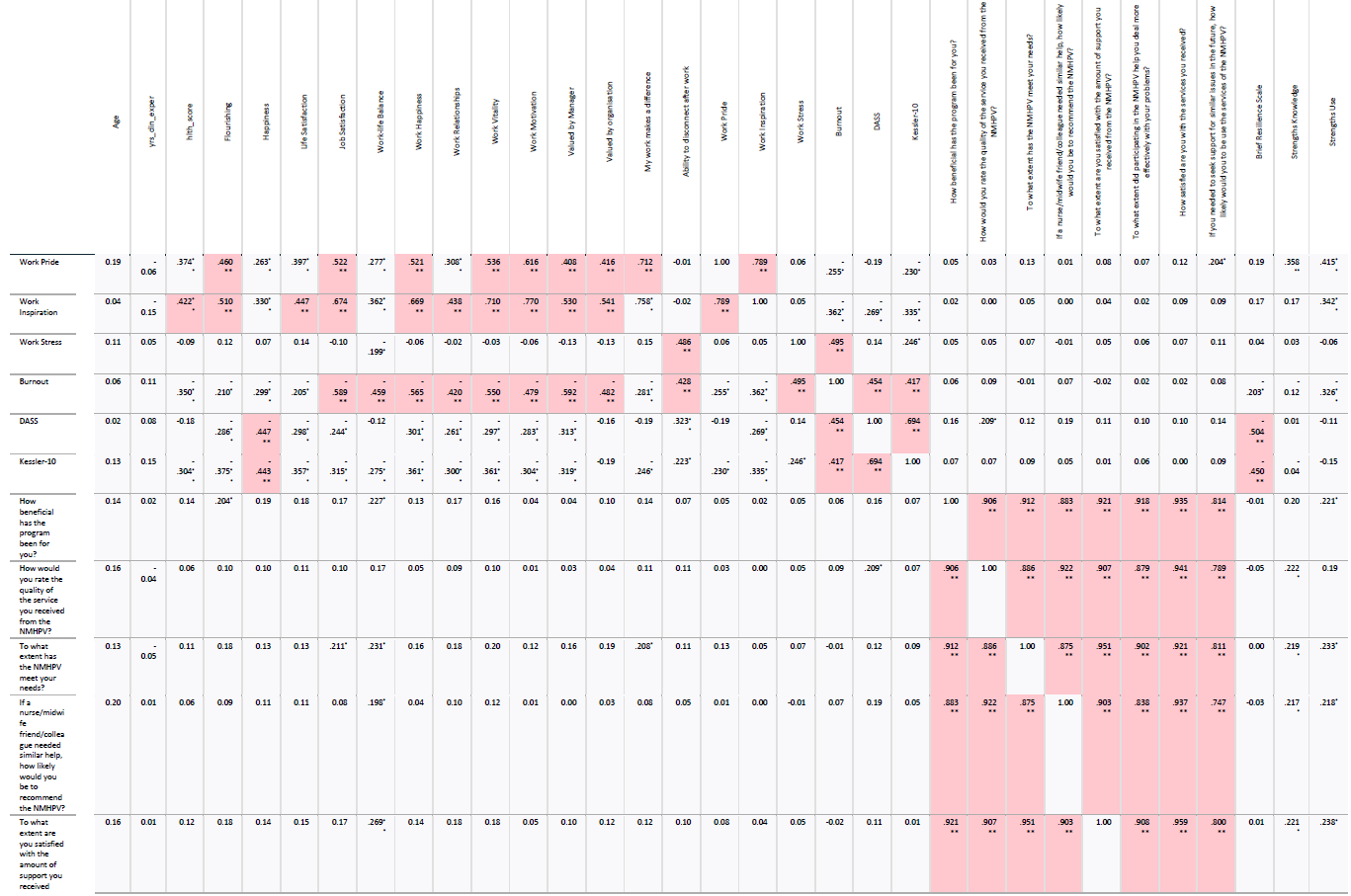


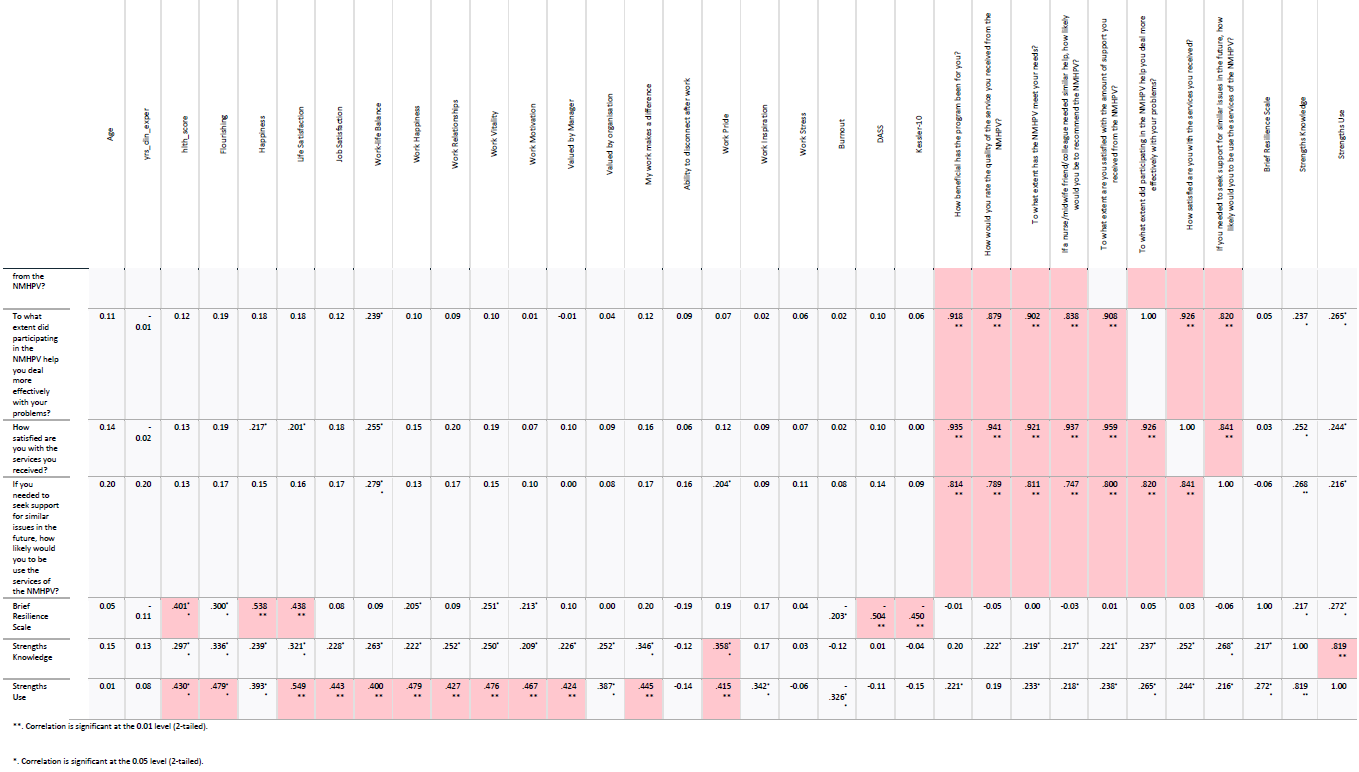


## Supplementary File 6. Summary of Program participant comments in response to the question “what are the three best things about the Program?”.

| Theme | Sub-theme | Illustrative comment(s) | Frequency of comments (*f*) | Alignment with program objectives |
| --- | --- | --- | --- | --- |
| Advice, Guidance, Support, Strategies |  |  |  | Develop and deliver services which promote case management and care coordination, and which are inclusive for diverse population groups, promote prevention, provide supportive responses and prioritise intervention and restoration of the individual’s health, within a health and wellbeing framework |
|  | Facilitated/inspired self-management strategies | *“I learnt some valuable thought patterns that I still use today.”*  *“The tools I now have in my head and written down to use in my daily life, to refer to when needed, knowing I'm ok.”* | 12 |  |
|  | Strategies and tools | *“practical assistance, not just analysis!”*  *“Constructive plan moving forward.”* | 27 |  |
|  |  |  |  |  |
|  | Support and help | *“Excellent advice and support”*  *“Brilliant facilitator who really gave me some Insight into my situation and amazing support”* | 35 |  |
| Being listened to, being heard, being understood, being validated |  | *“Understanding. My first consultant was mental health nurse. She fully understood my situation.”*  *“I could vent and cry and felt listened and understood.”* | 69 | Provide peer-based support services that are person-centred, evidence based, easily accessible through technology and sensitive to the health needs of the nursing and midwifery community, including prioritising those in susceptible and marginalised groups within the professions. |
| By nurses and midwives, for nurses and midwives |  | *“The counsellor had a nursing background herself so she was able to really understand the context unlike other counsellors.”*  *“The opportunity to discuss workplace issues with people who have share the lived experience of health professionals.”* | 34 | Provide peer-based support services that are person-centred, evidence based, easily accessible through technology and sensitive to the health needs of the nursing and midwifery community, including prioritising those in susceptible and marginalised groups within the professions. |
| Group discussions or activities |  | *“Group discussion about important discussion points such as the nursing standards”*  *“Meeting others who were struggling”* | 7 | Provide peer-based support services that are person-centred, evidence-based, easily accessible through technology and sensitive to the health needs of the nursing and midwifery community, including prioritising those in susceptible and marginalised groups within the professions. |
| Kind, compassionate, caring, friendly and warm |  | *“Friendly empathetic person at the end of the phone”*  *“Kindness Empathy and understanding”* | 22 | Develop and deliver services which promote case management and care coordination, and which are inclusive for diverse population groups, promote prevention, provide supportive responses and prioritise intervention and restoration of the individual’s health, within a health and wellbeing framework. |
| Non-judgemental |  | *“The willingness to listen and not judge and acknowledge the difficulties I was experiencing in a toxic workplace”*  *“The person I spoke to let me tell my story without judgment.”* | 14 | Provide peer-based support services that are person-centred, evidence-based, easily accessible through technology and sensitive to the health needs of the nursing and midwifery community, including prioritising those in susceptible and marginalised groups within the professions. |
| Practical features |  |  |  |  |
|  | **Availability / timeliness** | *“how very responsive to my request for support in a timely manner”* | 11 |  |
|  | **Confidential** | *“Someone confidential to help me work through my feelings, and experiences at work.”* | 3 |  |
|  | **Continuity with clinician** | *“Continuity with provider”* | 6 |  |
|  | **Ease of access** | *“That I can get any contact at any stage, I don't need a referral or specific health condition to participate.”* | 10 |  |
|  | **Free** | *“The service is free so when money is an issue you are not having to payout substantial amount of money”* | 13 |  |
|  | **Logistics** | *“Availability and flexibility re location, times, mode (in person or via phone if needed)”* | 7 |  |
|  | **Environment** | *“Low key but inviting...non-clinical”* | 2 |  |
| Safe |  | *“Having a safe space to discuss metal health issues”*  *“The program allowed me to voice my worries and issues in a completely safe environment”* | 6 | Develop and deliver services which promote case management and care coordination, and which are inclusive for diverse population groups, promote prevention, provide supportive responses and prioritise intervention and restoration of the individual’s health, within a health and wellbeing framework. |
| Independent/separate from work |  | *“Able to talk to someone uninvolved with work”* | 3 |  |
| Skilled professionalism |  | *“knowledge and professionalism of staff members was exceptional”* | 19 | Maintain service policies, procedures, and guidelines which promote and sustain diversity, inclusion, and equity, in keeping with the diverse needs of our consumers and professional standards. |
| Successful outcome |  | *“I got myself out of a bad situation and went on to get a new job that I enjoyed.”* | 9 | Develop and deliver services which promote case management and care coordination, and which are inclusive for diverse population groups, promote prevention, provide supportive responses and prioritise intervention and restoration of the individual’s health, within a health and wellbeing framework. |
| The clinician |  | *“Connection I made with the counsellor that I saw”* | 9 | To provide peer-based support services that are person-centred, evidence-based, easily accessible through technology and sensitive to the health needs of the nursing and midwifery community, including prioritising those in susceptible and marginalised groups within the professions. |

## Supplementary File 7. Referral pathways to and from the Program.

|  |  |  | Program Participant prior to 2020  (N=84) | | Program Participant in 2020 or later  (N=31) | | Total sample  (N=115) | |
| --- | --- | --- | --- | --- | --- | --- | --- | --- |
|  |  |  | N | % | N | % | N | % |
| Referral Pathways | Self-referred |  | 52 | 62 | 20 | 64% | 72 | 62% |
|  | Work referral |  | 7 | 8 | 4 | 13% | 11 | 10% |
|  | Cannot remember |  | 6 | 7 | 1 | 3% | 7 | 6% |
|  | Other |  | 19 | 23 | 6 | 19% | 25 | 22% |
|  | Union |  | 10 |  | 4 |  | 14 |  |
|  | AOD Support Service |  | 1 |  | 0 |  | 1 |  |
|  | Education institution |  | 1 |  | 0 |  | 1 |  |
|  | Friend |  | 1 |  | 0 |  | 1 |  |
|  | GP |  | 2 |  | 0 |  | 2 |  |
|  | The Program |  | 1 |  | 2 |  | 2 |  |
|  | Psychiatrist |  | 1 |  | 0 |  | 1 |  |
|  | Nursing & Midwifery Board |  | 1 |  | 0 |  | 1 |  |
|  | Workshop |  | 1 |  | 0 |  | 1 |  |
| Referral to other services | Did your engagement with the Program lead you to seek different types of support or other services? | |  |  |  |  |  |  |
|  | Yes |  | 32 | 41 | 14 | 47 | 46 | 43 |
|  | No |  | 36 | 47 | 16 | 53 | 52 | 49 |
|  | Cannot remember |  | 9 | 12 | 0 | 0 | 9 | 8 |
|  | Missing |  | 7 | - | 1 | - | 8 | - |
|  | Please describe the other services you accessed^b, c^ | |  |  |  |  |  |  |
|  | Psychologist/psychiatrist |  | 17 | 53 | 7 | 50 | 24 | 52 |
|  | GP |  | 7 | 22 | 2 | 14 | 9 | 20 |
|  | AOD Support |  | 3 | 9 | 0 | 0 | 3 | 6 |
|  | Union |  | 2 | 6 | 2 | 14 | 4 | 9 |
|  | EAP/Employment support |  | 2 | 6 | 1 | 7 | 3 | 6 |
|  | Online resources |  | 2 | 6 | 1 | 7 | 3 | 6 |
|  | Family support services |  | 2 | 6 | 1 | 7 | 3 | 6 |
|  | Legal services |  | 1 | 3 | 1 | 7 | 2 | 4 |
|  | Other |  | 2 | 6 | 1 | 7 | 3 | 6 |

*^a^Response options: 0 (Not at all) – 10 (Completely). ^b^Responses do not sum to number of people who responded to this question because some respondents reported accessing more than one service type. ^c^Expressed as percentage of respondents who reported accessing any service. Abbreviations” AOD = Alcohol and Other Drug, EAP = Employee Assistance Program, GP = General Practitioner.*

## Supplementary File 8. Goal setting as part of the program.

|  |  |  | Program Participant prior to 2020  (N=84) | | | | Program Participant in 2020 or later  (N=31) | | | | Total sample  (N=115) | | | |
| --- | --- | --- | --- | --- | --- | --- | --- | --- | --- | --- | --- | --- | --- | --- |
|  |  |  | N | | % | | N | | % | | N | | % | |
| Goal Setting | Did you set goals as part of your most recent involvement with the Program? | |  | |  | |  | |  | |  | |  | |
|  | Yes |  | 32 | | 42.1% | | 17 | | 56% | | 49 | | 45.5% | |
|  | No |  | 44 | | 57.9% | | 13 | | 44% | | 57 | | 54.5% | |
|  | (Missing) |  | (8) | | - | | (1) | | - | | (9) | | - | |
|  |  |  |  | |  | |  | |  | |  | |  | |
|  |  |  |  | | | |  | |  | |  | |  | |
|  |  |  | Mean (SD) | Range | | N | Mean (SD) | Range | | N | Mean (SD) | Range | | N |
|  | [if goals were set] To what extent do you feel that the goals you have set are achievable within the timeframes you have set?^a^ |  | 8.35 (2.20) | 0 – 10 | | 31 | 7.76 (1.85) | 4 – 10 | | 17 | 8.15 (2.08) | 0 – 10 | | 48 |
|  | To what extent do you feel like you are progressing with the goals you have set?^a^ |  | 8.16 (2.24) | 0 – 10 | | 32 | 7.53 (1.70) | 4 – 10 | | 17 | 7.94 (2.08) | 0 – 10 | | 49 |

*^a^Response options 0 (Not at all) – 10 (Completely). ^b^Responses do not sum to number of people who responded to this question because some respondents reported accessing more than one service type. ^c^Expressed as percentage of respondents who reported accessing any service.*

## Supplementary File 9. Features of the Program which influenced stakeholder decisions to refer to or recommend the program.

| Features influencing referral decision | N | Minimum | Maximum | Mean | Std. Deviation |
| --- | --- | --- | --- | --- | --- |
| Independent | 24 | 8 | 10 | 9.63 | .647 |
| Confidential | 24 | 8 | 10 | 9.54 | .721 |
| Does not require a formal referral | 24 | 6 | 10 | 9.17 | 1.167 |
| Designed and delivered by nurses / midwives | 24 | 7 | 10 | 9.13 | 1.035 |
| Free | 24 | 6 | 10 | 9.04 | 1.367 |
| Victorian state-wide service | 24 | 4 | 10 | 8.54 | 1.865 |
| Not time limited sessions | 24 | 0 | 10 | 7.79 | 2.874 |
| Observed impact of program on others | 24 | 1 | 10 | 7.63 | 2.779 |
| Quality Innovation Performance (QIP) accredited organisation | 24 | 0 | 10 | 5.83 | 3.002 |

*Abbreviation: QIP = Quality Innovation Performance*

Diener, E., Wirtz, D., Tov, W., Kim-Prieto, C., Choi, D. W., Oishi, S., & Biswas-Diener, R. (2010). New well-being measures: Short scales to assess flourishing and positive and negative feelings. *Social Indicators Research*, *97*(2), 143-156. <https://doi.org/10.1007/s11205-009-9493-y>

Govindji, R., & Linley, P. A. (2007). Strengths use, self-concordance and well-being: Implications for strengths coaching and coaching psychologists. *International Coaching Psychology Review*, *2*(2), 143-153.

Kessler, R., Barker, P., Colpe, L., Epstein, J., Gfroerer, J., Hiripi, E., Howes, M., Normand, S., Manderscheid, R., Walters, E., & Zaslavsky, A. (2003). Screening for serious mental illness in the general population. *Archives of General Psychiatry*, *60*(2), 184-189.

Kristensen, T., Hannerz, H., Høgh, A., & Borg, V. (2005). The Copenhagen Psychosocial Questionnaire-a tool for the assessment and improvement of the psychosocial work environment. *Scandinavian journal of Work, Environment Health & Social Care in the Community*, 438-449.

Lovibond, S., & Lovibond, P. (1996). *Manual for the depression anxiety stress scales* (2nd ed.). Psychology Foundation of Australia.

Schaufeli, W. B., Bakker, A. B., & Salanova, M. (2006). The measurement of work engagement with a short questionnaire: A cross-national study. *Educational and Psychological Measurement*, *66*(4), 701-716. <https://doi.org/10.1177/0013164405282471>

Smith, B., Dalen, J., Wiggins, K., Tooley, E., Christopher, P., & Bernard, J. (2008). The brief resilience scale: Assessing the ability to bounce back. *International Journal of Behavioral Medicine*, *15*(3), 194-200. <https://doi.org/10.1080/10705500802222972>
